# Identifying uncertainty in physical–chemical property estimation with IFSQSAR

**DOI:** 10.1186/s13321-024-00853-w

**Published:** 2024-05-30

**Authors:** Trevor N. Brown, Alessandro Sangion, Jon A. Arnot

**Affiliations:** 1 ARC Arnot Research & Consulting, Toronto, ON M4C 2B4 Canada; 2 Department of Physical and Environmental Sciences, University of Toronto Scarborough, Toronto, ON M1C 1A4 Canada; 3 Department of Pharmacology and Toxicology, University of Toronto, Toronto, ON M5S 1A8 Canada

**Keywords:** QSPR, PPLFER, Abraham solvation model, Octanol–water partitioning, Henry’s Law constant, Solubility, Vapor pressure, Prediction uncertainty, Applicability domain, Chemical properties

## Abstract

This study describes the development and evaluation of six new models for predicting physical–chemical (PC) properties that are highly relevant for chemical hazard, exposure, and risk estimation: solubility (in water *S*_*W*_ and octanol *S*_*O*_), vapor pressure (*VP*), and the octanol–water (*K*_*OW*_), octanol–air (*K*_*OA*_), and air–water (*K*_*AW*_) partition ratios. The models are implemented in the Iterative Fragment Selection Quantitative Structure–Activity Relationship (IFSQSAR) python package, Version 1.1.0. These models are implemented as Poly-Parameter Linear Free Energy Relationship (PPLFER) equations which combine experimentally calibrated system parameters and solute descriptors predicted with QSPRs. Two other ancillary models have been developed and implemented, a QSPR for Molar Volume (*MV*) and a classifier for the physical state of chemicals at room temperature. The IFSQSAR methods for characterizing applicability domain (AD) and calculating uncertainty estimates expressed as 95% prediction intervals (PI) for predicted properties are described and tested on 9,000 measured partition ratios and 4,000 *VP* and *S*_*W*_ values. The measured data are external to IFSQSAR training and validation datasets and are used to assess the predictivity of the models for “novel chemicals” in an unbiased manner. The 95% PI intervals calculated from validation datasets for partition ratios needed to be scaled by a factor of 1.25 to capture 95% of the external data. Predictions for *VP* and *S*_*W*_ are more uncertain, primarily due to the challenges in differentiating their physical state (i.e., liquids or solids) at room temperature. The prediction accuracy of the models for log *K*_*OW*_, log *K*_*AW*_ and log *K*_*OA*_ of novel, data-poor chemicals is estimated to be in the range of 0.7 to 1.4 root mean squared error of prediction (RMSEP), with RMSEP in the range 1.7–1.8 for log *VP* and log *S*_*W*_.

**Scientific contribution**

New partitioning models integrate empirical PPLFER equations and QSARs, allowing for seamless integration of experimental data and model predictions. This work tests the real predictivity of the models for novel chemicals which are not in the model training or external validation datasets.

**Graphical Abstract:**

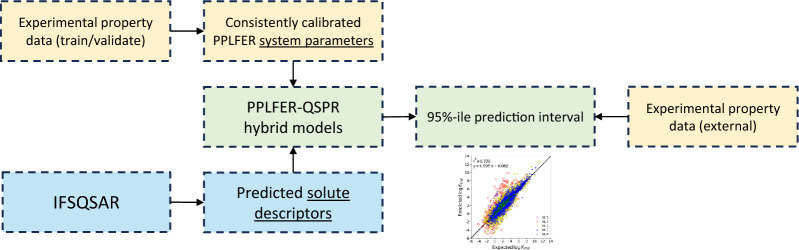

**Supplementary Information:**

The online version contains supplementary material available at 10.1186/s13321-024-00853-w.

## Introduction

Physical–chemical (PC) property data are essential for conducting legislated ecological and human health assessment for new and existing organic chemicals [1–3]. Common PC properties used in chemical assessments are solubility in water (*S*_*W*_; mol/L), solubility in octanol (*S*_*O*_; mol/L), vapor pressure (*VP*; Pa), melting point (*T*_*M*_; K), boiling point (*T*_*B*_; K) and the octanol–water (*K*_*OW*_), octanol–air (*K*_*OA*_), and air–water (*K*_*AW*_) partition ratios. The partition ratios are considered dimensionless, and *K*_*AW*_ is the dimensionless Henry’s Law Constant (*H*; Pa.m^3^/mol) as *K*_*AW*_ = *H*/*RT*, where *R* is the Ideal Gas Law Constant (Pa.m^3^/(mol.K)) and *T* is the system temperature (K; kelvin). Models used for predicting bioaccumulation [4], overall persistence and long-range transport potential [5], toxicity, toxicokinetics in in vitro and in vivo systems, chemical concentrations in natural and manufactured environments, and ultimately exposure to human and ecological receptors require at least some of the listed PC properties as input parameters. Chemical assessment outcomes are sensitive to the selected PC values, e.g., [5–9] and reliable PC data are therefore required for reliable chemical assessments; “garbage in = garbage out” [10]. There is a need to better understand which chemicals and properties have the greatest uncertainties so these sources of error in regulatory decision-making can be addressed.

Uncertainty in PC data is inherent whether the data are measured or modelled [11, 12] and guidance for selecting PC data for chemical assessments is available [11]. Theoretical relationships between *S*_*W*_, *S*_*O*_, *VP*, *K*_*OW*_, *K*_*OA*_, and *K*_*AW*_ have been outlined by Mackay and colleagues [13–15] and others [16, 17]. These theoretical relationships (sometimes referred to as the “three solubility approach” [15]) can be applied for evaluating measured and predicted PC property data quality and obtaining consistency amongst them all as a method to address uncertainty in available PC property data and guide the selection of reliable data. Predictive methods for PC property data are required for thousands of chemicals legislated for evaluation [18–21]. Methods for predicting PC properties include Quantitative Structure-(Activity)Property Relationships (QS(A)PRs) and Poly-Parameter Free Linear Energy Relationship (PPLFER), also known as Abraham equations [22, 23]. Organization for Economic Co-operation and development (OECD) guidance for QS(A)PR development and validation for applications in regulatory decision-making exists [24, 25] including consideration of the applicability domain (AD) for a predicted property as outlined in the recent OECD QSAR assessment framework (QAF) [26]. There is a need for reliable predictive methods that include AD information as well as uncertainty estimates for predictions.

The Iterative Fragment Selection QSAR (IFSQSAR) development methods have been progressively updated and applied to various chemical properties over the last 10 years [27–29]. IFSQSARs are fragment-based multiple linear regression (MLR) models developed using extensive cross-validation and conservative goodness-of-fit metrics to create robust and predictive models, and make predictions based only on the chemical structure as a Simplified Molecular Input Line Entry System (SMILES) string [30]. The IFSQSARs include the prediction of solute descriptors required to parameterize PPLFER equations and other PC properties directly. The IFSQSARs have been developed in agreement with OECD guidance and apply three complementary methods for assessing if predictions are within the QSPR AD and provide estimates of the prediction uncertainty. The IFSQSAR methods and the mechanistic insights of the PPLFER methods are applied in this work to identify and characterize general uncertainties in predicting PC property data required for chemical assessments. The model development ensures that predicted properties are thermodynamically consistent, and their calculation is based on a consistent set of descriptors, i.e. the PPLFER solute descriptors. This is like previous efforts based on different descriptors, such as the Unified Physicochemical Property Estimation Relationships (UPPER) method of Yalkowsky and colleagues [31].

The present study describes the development and evaluation of new models in IFSQSAR Ver.1.10 (https://github.com/tnbrowncontam/ifsqsar) for predicting *S*_*W*_, *S*_*O*_, *VP*, *K*_*OW*_, *K*_*OA*_, and *K*_*AW*_. The new models, and other QSARs, are available in a user-friendly, freely accessible online platform, the Exposure And Safety Estimation (EAS-E) Suite (www.eas-e-suite.com). QSPRs have previously been developed for solute descriptors and system parameters of PPLFERs [32, 33]. These QSPRs are combined with empirically calibrated PPLFER equations to make predictions for PC properties, some calibrated in previous research [34] and some newly calibrated in this work. A key objective of this work is to validate the predictive power of the new models against experimental data for novel chemicals; therefore, in the validation process, the PPLFERs are only parameterized with solute descriptors predicted by the IFSQSARs to represent conditions of applying models to chemicals and properties for which there are no measured data. The new model predictions are compared against independent measured property data to assess their predictive power (uncertainty) expressed as 95% prediction intervals. Methods for quantifying the predictive power of the QSPR predictions for novel chemicals, i.e. chemicals that are outside of the training and validation datasets, are evaluated. Based on these evaluations and the detailed AD information of the IFSQSAR models, methods for further improving the understanding of the prediction uncertainty for novel chemicals are recommended.

## Methods

### Theory

**Fig. 1:**
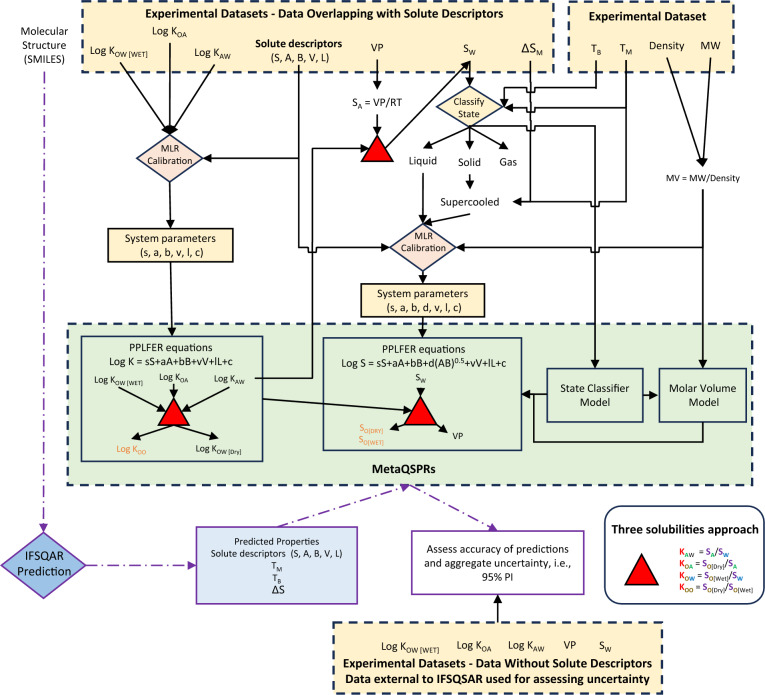
Schematic of the workflow in this research. Yellow boxes represent experimental data and empirical models, blue boxes represent QSPR predictions, green boxes represent MetaQSPRs which combine both, orange text represents models calibrated only by thermodynamic property cycle, purple arrows represent the validation process in which only the chemical structure (SMILES) is used to apply the models. log *K*_*OW*_, log *K*_*OA*_, log *K*_*AW*_ partition ratios, *VP*, *S*_*A*_ vapor pressure and solubility in air, *S*_*W*_ solubility in water, *S*_*O*_ solubility in octanol, MLR multiple linear regression, *ΔS* entropy of melting, *T*_*B*_ boiling point, *T*_*M*_ melting point, *MW* molecular weight, *MV* molar volume

Thermodynamic property cycles that describe the interrelation between partitioning and solubility in octanol, water and air phases are referred as the three-solubility approach. The three-solubility approach interprets the partition ratios *K*_*OW*_, *K*_*OA*_, and *K*_*AW*_ as ratios of the solubilities *S*_*O*_, *S*_*W*_ and solubility in air (*S*_*A*_), where *S*_*A*_ is a conversion of *VP* at atmospheric pressure and temperature. Figure 1 shows how the three-solubility approach [15] is used in this study to calibrate consistent solubility and partitioning properties. Partition ratios and solubility in this work are calculated using PPLFERs. PPLFERs were pioneered by Michael Abraham and colleagues, and are empirical correlations used to predict chemical properties with many applications in environmental chemistry [33]. There are three different forms of PPLFER equations which include different sub-sets of solute descriptors and system parameters. Two forms are recommended by Abraham for partitioning between two condensed phases, or partitioning between one condensed phase and one gaseous phase [22]. A third form was suggested by Goss [23] which contains descriptors from each of the two suggested by Abraham and is shown in Eq. 1. PPLFERs in the form of Eq. 1 are used in this work because they offer two advantages for environmental chemistry research. The first is that using a single form of the equation allows for the application of thermodynamic property cycles. The second is that this form of PPLFER equation shows better predictive power for some solutes with unique properties, including perfluorinated alkyl substances and methyl siloxanes, which are of environmental interest [35].

1 $${\text{log}}\;{\text{K}}{\mkern 1mu} = {\mkern 1mu} {\text{s}} \cdot {\text{S }}{\mkern 1mu} + {\mkern 1mu} {\text{a}} \cdot {\text{A }}{\mkern 1mu} + {\mkern 1mu} {\text{b}} \cdot {\text{B }}{\mkern 1mu} + {\mkern 1mu} {\text{v}} \cdot {\text{V}}{\mkern 1mu} + {\mkern 1mu} {\text{l}} \cdot {\text{L}}{\mkern 1mu} + {\mkern 1mu} {\text{c}}$$

PPLFER equations consist of solute descriptors, which correlate with the molecular interactions of the solute, and system parameters which are fitted to the properties of the system of interest. For partition ratios the system will be the two phases that the partition coefficient describes, and the system parameters describe the relative propensity for solutes to partition to one phase or the other with positive values favoring the first phase and negative values favoring the second phase. For solubility the two phases are the pure phase of the solute and water, air, or octanol. System parameters are determined by MLR of the property against experimentally determined solute descriptors of the dataset of training chemicals for which both the solute descriptors and property are available, this is referred to as calibrating a PPLFER equation. Experimental solute descriptors are available for about 8000 solutes and system parameters have been calibrated for solvent-air and solvent–water partitioning of about 100 solvents including octanol [36, 37].

In Fig. 1, Table 1, and Eq. 1 the lower-case letters *s*, *a*, *b*, *v*, *l*, and *c* are the system parameters specific to the system. The upper-case letters *S*, *A*, *B*, *V*, and *L* are the solute descriptors specific to the solute. For solubility an additional term that combines A and B with an additional system parameter *d* is required, as discussed below. The solute descriptors correlate with different types of molecular interactions: *S* is a combination of the solute dipolarity and polarizability, *A* is the hydrogen bond donor capacity, *B* is the hydrogen bond acceptor capacity, *V* is the McGowan volume which has been interpreted as correlating with energy of cavity formation, and *L* is the partition coefficient for the hexadecane-air system which correlates with van der Waals interactions. Abraham has also calibrated PPLFER equations for the pure phase properties solubility *S*_*W*_ [38] and vapor pressure *VP* [39]. Separate PPLFER equations were developed for liquid and solid solutes with quite different system parameters. These PPLFER equations represent a system where partitioning is between the chemical pure phase and the water and air phases meaning that the system is different for every solute which is not consistent with how PPLFERs are typically applied. Equation 2 shows a PPLFER equation analogous to Eq. 1 for solubility of solute in water, octanol, or air, which has been modified according to Abraham’s method  38, 39.

2 $${\text{logS}}_{{\text{[W,O,A]}}} \,{ = }\,{\text{s}} \cdot {\text{S }}\,{ + }\,{\text{d}} \cdot {\text{A}} \cdot {\text{B }}\,{ + }\,{\text{v}} \cdot {\text{V}}\,{ + }\,{\text{l}} \cdot {\text{L}}\,{ + }\,{\text{c}}$$

**Table 1:** Poly-Parameter Free Linear Energy Relationship (PPLFER) system parameters^a^

System	s	a	b	d^c^	v	l	c	Total s.e	References
log K_AW_	−2.26 (0.05)	−3.72 (0.04)	−4.78 (0.04)		2.19 (0.06)	−0.38 (0.02)	0.64 (0.03)	0.17	[34]
log K_OA_	0.69 (0.05)	3.56 (0.04)	0.73 (0.04)		0.52 (0.08)	0.79 (0.02)	−0.26 (0.03)	0.16	[34]
log K_OW_	−1.36 (0.04)	−0.13 (0.03)	−3.49 (0.03)		2.41 (0.06)	0.41 (0.01)	0.41 (0.03)	0.15	This work
dry log K_OW_	−1.57 (0.07)	−0.16 (0.06)	−4.05 (0.06)		2.71 (0.10)	0.41 (0.02)	0.38 (0.04)	0.23	^d^
log K_O[w]O[d]_	0.21 (0.08)	0.03 (0.06)	0.56 (0.06)		−0.30 (0.12)	0.00 (0.03)	0.03 (0.05)	0.28	This work^d^
log VP_[l]_ (Pa)	−1.55 (0.12)	−0.92 (0.23)	−0.63 (0.13)	−1.60 (0.27)	−1.30 (0.18)	−0.51 (0.05)	7.13 (0.08)	0.59	This work
log S_W[l]_ (mol/L)	0.71 (0.11)	2.80 (0.23)	4.15 (0.13)	−1.60 (0.27)	−3.49 (0.17)	−0.13 (0.04)	0.18 (0.07)	0.60	This work
log S_O[d][l]_ (mol/L)	−0.86 (0.13)	2.64 (0.23)	0.10 (0.14)	−1.60 (0.27)	−0.78 (0.20)	0.28 (0.05)	0.56 (0.08)	0.64	This work^d^
log S_O[w][l]_ (mol/L)	−0.65 (0.12)	2.67 (0.23)	0.66 (0.13)	−1.60 (0.27)	−1.08 (0.18)	0.28 (0.04)	0.59 (0.08)	0.62	This work^d^

^a^The standard error (s.e.) for each system parameter is shown in parentheses

^b^System parameter corresponding to solute descriptor E, excess molar refraction, not used elsewhere in this work

^c^System parameter corresponding to the term (*A*∙*B*)^0.5^

^d^System parameters calculated by thermodynamic property cycle. Total s.e. and s.e. of the coefficients are estimated by propagation of uncertainty

In these PPLFERs the solute descriptors are being used to describe how a chemical behaves as both a solute and the solvent. The *A*∙*B* term explicitly accounts for the effects of hydrogen bonding between molecules of the chemical, and some versions proposed by Abraham [39, 40] include an *S*∙*S* term to account for dipole–dipole interactions. The system parameters quantify how each solute descriptor favors solubility in water, octanol, or air, and any broadly applicable interactions within the pure phase of the solute. Equation 2 was modified to Eq. 3 in this work, because this was found to give better fitting results, and the (*AB*)^0.5^ term is more consistent with previous work done predicting system parameters [34]:

3 $${\text{logS}}_{{\text{[W,O,A]}}} \,{ = }\,{\text{s}} \cdot {\text{S}}\,{ + }\,{\text{a}} \cdot {\text{A}}\,{ + }\,{\text{b}} \cdot {\text{B}}\,{ + }\,{\text{d}} \cdot \left( {{\text{A}} \cdot {\text{B}}} \right)^{{{0}{\text{.5}}}} \,{ + }\,{\text{v}} \cdot {\text{V}}\,{ + }\,{\text{l}} \cdot {\text{L}}\,{ + }\,{\text{c}}$$

Previous research developed empirical regressions between solute descriptors and system parameters for solvent-air partitioning which can be used as an alternative method to predict solubility [34]. System parameters of PPLFER equations in the form of Eq. 1 can be predicted for each solute using the empirical regressions. These predicted PPLFER equations are then used to predict the partitioning of a solute between air and the solute’s own pure liquid phase, giving a partition ratio (log *K*_*kAk*_). These log *K*_*kAk*_ values are then converted to *VP* using Eq. 4, which is a rearrangement of Raoult’s Law [34], and converted to *S*_*W*_ by the three-solubility approach. In Eq. 4*γ* is the activity coefficient of the solute which is assumed to be unity in the pure phase, and *MV* is the molar volume of the liquid or supercooled liquid solute. *VP* is then unit converted to *S*_*A*_ at standard temperature and pressure and a thermodynamic property cycle is applied to calculate *S*_*W*_ and *S*_*O*_ from the calibrated PPLFER equations for log *K*_*AW*_ and log *K*_*OA*_.

4 $${\text{logVP}}\,{ = }\,{\text{log}}\left( {\frac{{{\text{RT}}}}{{{\gamma K}_{{{\text{kAk}}}} {\text{MV}}}}} \right)$$

This indirect method has only been validated for predicting the *VP* of liquids, and testing done in this work for solids showed that the results were poor.

PPLFER equations for partition ratios involving pure solvent phases, water, and air typically have standard errors of fitting and prediction of less than 0.2 log units when calibrated with experimental solute descriptors. The Abraham PPLFERs for have larger errors on the order of 0.3 log units for liquids and up to 0.8 log units for some solids, but these equations also contain other correction factors for specific functional groups [39]. For *S*_*W*_ the error is about 0.6 log units [38]. The indirect method for calculating solubilities had errors of about 0.4 and 0.5 log units when applied to solubility in air for liquids. All these statistics are calculated on different datasets and are typically fitting errors rather than predictive errors, so they give an idea of the goodness of fit of the models, but not necessarily the predictive power. If PPLFERs are properly calibrated with sufficient data then they have broad applicability and accuracy [35].

Table 1 summarizes the PPLFER equations used in this work to predict PC properties. The equations for log *K*_*OA*_, and log *K*_*AW*_ have been calibrated in previous work [32, 34], the system parameters for dry log *K*_*OW*_ (pure octanol) are calculated as the sum of the system parameters for log *K*_*OA*_ and log *K*_*AW*_, i.e., using the three solubility approach. Sections SI-2, SI-3, and SI-4 detail the calibration of new PPLFER equations in this work, for wet log *K*_*OW*_ (water saturated octanol), log *K*_*OO*_ (hypothetical partition ratio between wet and dry octanol), *VP*, *S*_*W*_, and *S*_*O*_ (dry and wet). One of the goals of this work is to create models that predict partition ratios and solubilities which have thermodynamic consistency built in, and this is achieved by calibrating the PPLFER system parameters to be thermodynamically consistent using the concept of the three solubility approach [15]. The PPLFER equations in this work have all been calibrated on experimental data except for *S*_*O*_, which is only calculated by the three solubility approach due to limited data availability and is shown in a different color in Fig. 1 to reflect this.

One challenge in this process is that there is an inherent discrepancy in the three solubility approach with regards to how the data are measured. Most measurements of log *K*_*OW*_ are performed with the octanol and water phases in direct contact so that the octanol becomes saturated with water and vice versa. The solubility of octanol in water is very low so the effect of partitioning of chemicals to the water phase is negligible. However, a significant amount of water is soluble in the octanol phase, and this changes the partitioning properties [41]. The PPLFER system parameters in Table 1 show the “dry” log *K*_*OW*_ will be lower than the “wet” log *K*_*OW*_ for polar and hydrogen bonding chemicals because the *s*, *a*, and *b* system parameters are lower. In contrast, log *K*_*OA*_ measurements are usually made using dry octanol [42]. In addition, the difference between wet *S*_*O*_ (*S*_*O[w]*_) and dry *S*_*O*_ (*S*_*O[d]*_) must be considered. A PPLFER for a hypothetical partition ratio between wet and dry octanol (*K*_*OO*_) has been derived in this work which can make these corrections, ensure thermodynamic consistency, and is implemented as a QSPR in IFSQSAR.

### IFSQSAR description and AD

The IFSQSAR development methods have been described in previous work [27–29, 32, 43] and are summarized in Section SI-1. An important aspect to understand for this work is the division of experimental data into a training dataset used to calibrate the QSPR and a validation dataset used to validate the QSPR and estimate the prediction uncertainty. The splitting is rational and deterministic, ensuring that both datasets represent the chemical diversity of the experimental data and the range of expected values. The solute descriptor QSPRs were trained and validated on a common dataset, so that each solute is only in either the training or validation dataset for all solute descriptor QSPRs. Further details on the dataset splitting are in Brown 2022 [32]. All the QSPRs and PPLFERs described here are coded in the IFSQSAR version 1.1.0 python package and implemented in the EAS-E Suite online platform (www.eas-e-suite.com). IFSQSARs apply three complementary approaches to define the basic AD of predictions, the first two approaches are very similar to, but developed in parallel to the AD methods applied by OPEn structure–activity/property Relationship App (OPERA) [44]. The first approach uses the leverage which is interpreted as a measure of extrapolation from the training dataset [45, 46], and the second is Chemical Similarity Score (CSS) which is a nearest neighbours approach and is less sensitive to extrapolation. Various cut-offs are defined for both approaches and are combined to assign each QSPR prediction an Uncertainty Level (UL) between UL 0–3 which correlates with uncertainty of the QSPR predictions, or inversely correlates with predictive power. Individual predictions can always be good or bad regardless of the UL, the UL only quantifies the typical uncertainty. Some special cases are also defined, UL 4 means that all fragments in the QSPR have a count of zero for the chemical, this may be a defined as in or out of the AD depending on the meaning of the intercept. UL 5 is the third complementary AD approach and has been described as a “denylist” AD check [47], but also might be described as a negative domain check, or inverse structural alerts. All the information about atoms and bonds in the training dataset is summarized regardless of whether the exact substructures are included in the fragments selected for the QSPR. Chemicals are checked against this summary and if they contain a substructure that is not found in the training data then they are flagged as UL 5. Finally, for some QSPRs it is pragmatic to set boundary conditions on possible values, and any predictions which violate these boundary conditions are flagged as UL 6. Table 2 summarizes the seven IFSQSAR ULs.

**Table 2:** IFSQSAR uncertainty level (UL) specifications

UL	Description
UL 0	In the AD, no warnings by leverage or CSS
UL 1	In the AD, borderline case warning by leverage or CSS
UL 2	Out of AD, warning by leverage or CSS
UL 3	Out of AD, egregious extrapolation warning by leverage
UL 4	In or out of AD, prediction is just the intercept, depends on meaning of the intercept
UL 5	Out of AD, uncalibrated atom or bond types, prediction may be wrong in unpredictable ways
UL 6	Out of AD, prediction is outside min/max of a bounded property

The IFSQSARs that use chemical structure to predict solute descriptors (used in PPLFER equations) and other PC properties directly provide an UL and predictivity metric along with each prediction [32]. Here predictivity refers to the predictive power of the QSPR, i.e. how accurate the predictions are likely to be, or inversely how uncertain the predictions are likely to be. Predictivity is quantified by the root mean squared error of prediction (RMSEP) as calculated from the external validation dataset of each solute descriptor QSPR, more discussion of the RMSEP can be found in “Metrics of model performance and predictivity” section. As the RMSEP increases the predictivity is lower and the uncertainty is higher.

All the property PPLFER equations in IFSQSAR are implemented as Meta QSPRs. Meta QSPRs use the outputs of other QSPRs as their inputs and calculate new values, aggregated ULs, and error estimates. For example, log *K*_*OW*_ is estimated with a Meta QSPR which combines solute descriptors predicted by QSPR and the experimental system parameters from Table 1 in PPLFER Eq. 1. All the PPLFER equations in this work (*K*_*OW*_, *K*_*AW*_, *K*_*OA*_, *VP*, *S*_*W*_ and *S*_*O*_) are implemented as Meta QSPRs. Note that IFSQSAR will by default use experimental solute descriptors instead of predicted ones where possible to increase the accuracy of predictions. This feature of IFSQSAR was not included in the validation process of this study so that only predicted solute descriptors were used to evaluate the models’ expected predictivity for novel or data-poor chemicals. The AD and predictivity as UL and RMSEP of the Meta QSPRs are calculated as an aggregate of UL and RMSEP of the Meta QSPR model inputs and other parameters written into the model such as the experimental system parameters. The details are described elsewhere [32], but in brief the aggregated UL and RMSEP are calculated according to propagation of uncertainty rules. These calculations are done automatically in the Meta QSPR code and documented in the output.

Meta QSPRs for predicting *VP* and *S*_*W*_ for liquids have already been implemented in previous work based on QSPRs that predict the PPLFER system parameters for liquid solvents [32]. These are referred to as indirect predictions in the present study as opposed to the direct predictions of *VP* and *S*_*W*_ made with the new PPLFER system parameters in Table 1. As outlined in “Model evaluations with empirical datasets and endpoint relevance” section it is known that *VP*, *S*_*W*_ and *S*_*O*_ for liquids and solids have notable differences. To help account for these differences two previously created QSPRs were used, and two new ones were created. The previously developed direct prediction QSPRs are the entropy of fusion (*ΔS*_*M*_) and *T*_*M*_ [29]. The first new QSPR introduced in this study is a new classifier model to predict whether a chemical is a gas, liquid or solid at 25 °C and standard atmospheric pressure to predict when corrections for solids need to be applied. The state classifier is implemented as a Meta QSPR which takes solute descriptors, *T*_*M*_, and *T*_*B*_ as inputs, and is described in Section SI S-5. Finally, as discussed in “Model evaluations with empirical datasets and endpoint relevance” section the values for *S*_*W*_ and *S*_*O*_ are capped at solute molar volume (*MV*) in some cases; therefore, Section SI-6 describes a new QSPR for *MV* developed in this study.

### Model evaluations with empirical datasets and endpoint relevance

Figure 1 shows the general workflow and the relationships between properties datasets and the models developed in this study. Yellow filled boxes represent experimental datasets, and in the case of the system parameters, values that have been empirically calibrated using only experimental data inputs. Blue filled boxes represent QSPR predictions, and green filled boxes represent hybrid models which combine QSPR predictions with system parameters calibrated on experimental data. There is a separate PPLFER equation and model for each property, but the calibration of the system parameters for all partitioning properties are interrelated through the three solubility approach. The main division of experimental data is solutes with available solute descriptors which is used from training and validating the models (top left box), and solutes with partitioning data but no solute descriptors (bottom box). IFSQSAR predictions were made for the following PC properties then evaluated using datasets of experimental values originally from the PhysProp database included in EPI Suite package [48]: log *K*_*OW*_, log *K*_*AW*_, log *K*_*OA*_, log *VP*, and log *S*_*W*_. These predictions and data are then used to assess the predictivity of IFSQSAR PPLFER-based models for novel chemicals. The PhysProp datasets have been further curated as a part of the creation of the OPERA QSAR package, including assigning all chemicals QSAR-ready structures as SMILES [44, 49]. Chemicals have been matched by CAS number with chemicals in the solute descriptor database used to develop the IFSQSARs [32], and identified as being in the training dataset, the validation dataset, or in neither. Chemicals in neither dataset are novel and are referred to here as being external to IFSQSAR.

There are several caveats to consider when comparing the IFSQSAR model predictions to the experimental datasets of PC properties. The first thing to consider is the difference between wet and dry octanol, as described in “Theory” section. Secondly, PC properties involving a pure chemical phase such as *VP* and *S*_*W*_ are different for liquids and solids. Chemical fate and transport models typically assume that all chemicals are liquids, or supercooled liquids, also called subcooled liquids. The theory is that at very low concentrations in a phase the solid chemicals behave as liquids because there are never enough molecules to form a solid pure phase. Measured or predicted *VP* and *S* data for solids can be corrected to equivalent supercooled liquid values using the Clausius Clapeyron equation or one of its simplifications, the most common being the Van’t Hoff approximation [50]. This is discussed in more detail in Section SI-4. As discussed in the previous section the data inputs required to apply the Van’t Hoff approximation, *ΔS*_*M*_ and *T*_*M*_, were developed in previous work, and the new classifier helps determine if a chemical is likely to be a liquid or a solid at system temperature (default in IFSQSAR = 25 °C).

Another end point mismatch that is commonly encountered in partitioning data is the partitioning of ions and ionizable chemicals. This is mostly important for partitioning where water is one of the phases, although the effect in other phases, e.g., water-saturated octanol, is possible. The present study only focusses on the partitioning of neutral organic chemicals. Chemical ionization is only considered in this work to identify experimental data where the measurement may be influenced by it and remove those data from model development and evaluation. Strong acids and bases are identified as acids with a *pK*_*a*_ less than 4 and bases with a *pK*_*a*_ greater than 10 and were removed. Experimental *pK*_*a*_ were collected from the curated OPERA database. If a *pK*_*a*_ was not available, a consensus value between ChemAxon estimates (available in the ChEMBL database [51]) and ACD Labs 2023.1.0 (Build 3666) was determined.

In this study upper boundaries have been set for *VP* and *S*_*O*_ and *S*_*W*_ values. When a solute is miscible in water or octanol there is no limit for how much of the solute can be dissolved. This might be expressed as a *S* where the amount of the solute is greater than the amount of water or octanol, which is not measurable or physically reasonable in a real system. We propose as a reasonable upper boundary on all solubility values to use the inverse of the solute liquid *MV*, i.e., the concentration of solute in its own pure liquid phase. The liquid *MV* QSPR developed in this work is used to set the capped value for solubility predictions. A similar upper boundary can be defined for *VP*, in this case we use standard atmospheric pressure as the upper boundary, because in the context of modelling the natural environment the pressure of a chemical will not be greater than this value.

### Metrics of model performance and predictivity

The RMSEP is calculated from experimental values of the external validation datasets and predicted values from IFSQSAR PPLFER based models using Eq. 5:

5 $${\text{RMSEP}}\,{ = }\,\left( {\frac{{\sum\nolimits_{{{\text{i}}\, = \,1}}^{{{\text{n}}_{{{\text{ext}}}} }} {\left( {{\text{y}}_{{\text{i}}} \, - \,\widehat{{\text{y}}}_{i} } \right)^{2} } }}{{{\text{n}}_{{{\text{ext}}}} }}} \right)^{0.5}$$

where n_ext_ is the number of data points in the validation dataset, y_i_ are the experimental values and ŷ_i_ are the predicted values. The RMSEP can is then used to calculate an estimated 95% prediction interval (PI) using Eq. 6:

6 $${95}\% \,{\text{PI}}\, = \,\left[ {{\text{M}}\,{-}\,{\text{RMSEP}}*{1}.{96},\,{\text{M}}\, + \,{\text{RMSEP}}*{1}.{96}} \right]$$

where M is the predicted PC property value. In an ideal case the validation dataset of a QSPR is representative of the structural diversity of chemicals to which the model might be applied. In this ideal case the RMSEP calculated from the validation dataset would be a good estimate of global RMSEP and 95% of predictions would have the experimental value contained within their PI. However, in practice the data available for validating QSPRs is limited by the experimental methods used to measure the data and will not be representative of the diversity of chemicals to which the model may be applied, so the RMSEP and the PI will only be estimates.

In cases where predictions are made for chemicals that are well within the AD, the RMSEP is typically comparable to the goodness-of-fit quantified as the standard deviation between the experimental and fitted value of the training dataset, i.e., the same as Eq. 5 but between the experimental values of the training dataset and the fitted QSPR values. The further out of the AD a group of predictions are, the larger the real RMSEP will be. As stated above, individual predictions can always be good or bad regardless of whether they are in the AD or not, the RMSEP is a probabilistic metric.

During IFSQSAR model development each chemical in the external validation dataset is assigned a UL as discussed in “IFSQSAR description and AD” section, and then the RMSEP is calculated for all chemicals within each UL. ULs 0 to 3 almost always have an increasingly large RMSEP for investigated datasets [29, 32, 43]. UL 4 may have a high or low RMSEP depending on if intercept-only predictions are considered within the AD, which depends on the property and structure of the model. Because UL 5 means that the chemical contains atoms or bonds not represented in the training dataset the RMSEP also cannot be reasonably estimated because the untrained atoms and bonds may have unexpected effects on the property. However, in practice the RMSEP of predictions for UL 5 has typically been comparable the RMSEP for UL3, provided that the chemicals are not inorganic. UL 6 means that the model has made a prediction outside of a boundary condition set at the time of model calibration. This UL is assigned after a normal prediction is made and an UL is assigned, the RMSEP of the original UL is used. The same trends of RMSEP with aggregate UL are observed for the PPLFER based models in this work.

One major goal of this work is to assess the accuracy of the RMSEP estimates provided by IFSQSAR models when compared to data that are not in the training or validation datasets, i.e., novel chemicals. The RMSEP values (in log units) will then be adjusted for the partitioning properties log *K*_*OW*_, log *K*_*AW*_, log *K*_*OA*_, *S*_*A*_ and *S*_*W*_ based this comparison. To do this PIs are calculated from the RMSEP and then the actual fraction of predictions within the PI are calculated to assess the accuracy of the PI and the RMSEP. The RMSEPs of each partitioning property are adjusted until the 95% PIs contain at least 95% of the experimental values, by multiplying by a factor increase depending on the trends observed for different ULs or chemical states.

## Results and discussion

### Evaluation of IFSQSAR partition ratio predictions

**Fig. 2:**
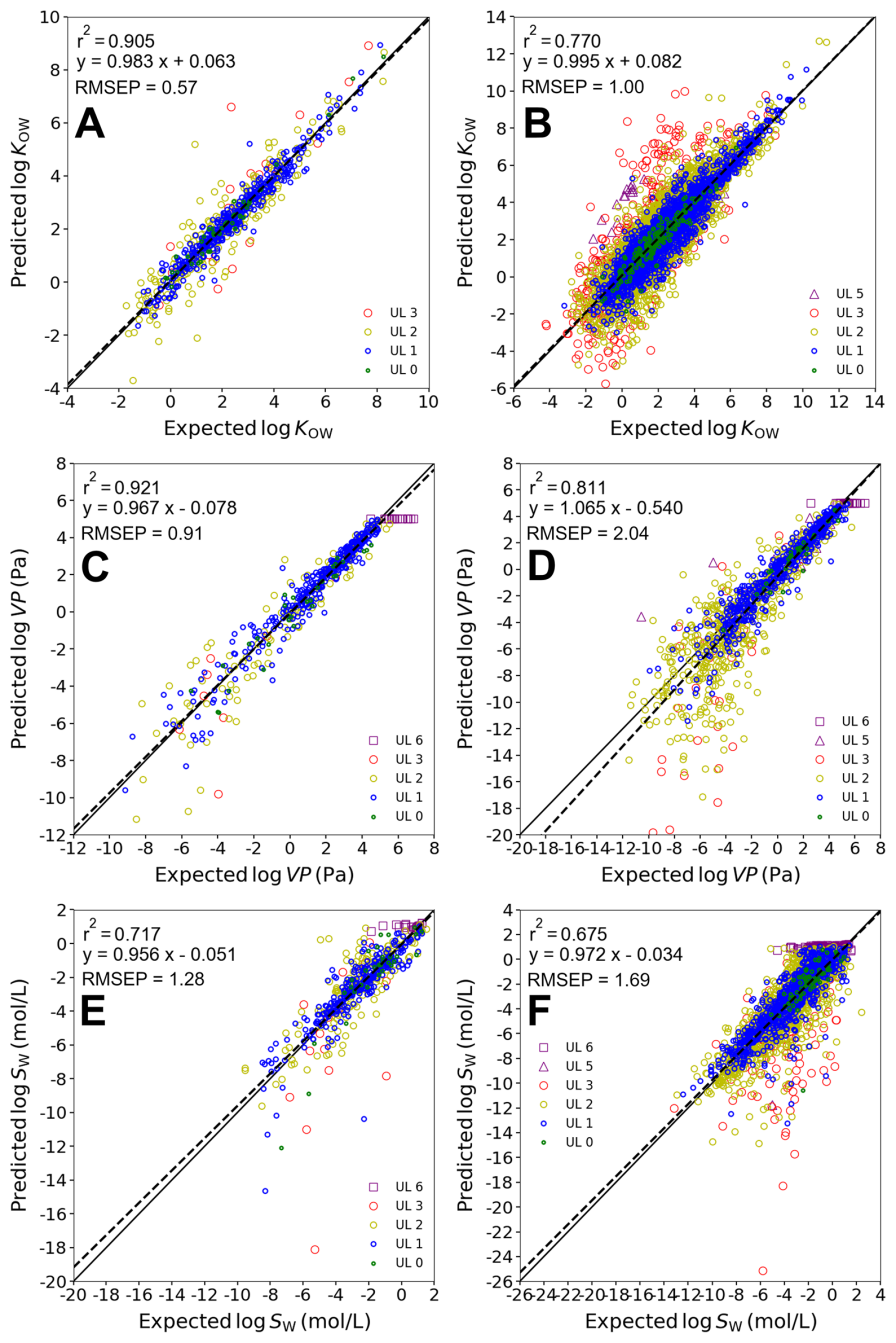
Comparisons of predicted and experimental data. **A** log *K*_*OW*_ of IFSQSAR validation set (n = 704) **B** log *K*_*OW*_ of external set (n = 8416) **C** log *VP* of IFSQSAR validation set (n = 495) **D** log *VP* of external set (n = 1207) **E** log *S*_*W*_ of IFSQSAR validation set (n = 529) **F** log *S*_*W*_ of external set (n = 2809)

Figures 2A, B show the IFSQSAR predictions compared to experimental *K*_*OW*_ data split into two subsets. Figure 2A includes chemicals that have experimental solute descriptors and are in the IFSQSAR validation dataset, but the plotted values are the IFSQSAR predictions. Figure 2B shows chemicals with no experimental solute descriptors, which are therefore entirely external to the IFSQSAR partition ratio and solubility models. The data points in Fig. 2 are colored by the aggregate UL of the predictions with UL 0, the least uncertain, colored green and UL 1 to UL 3 colored blue, yellow and red, which corresponds to increasing uncertainty. Data points with lower UL tend to fall closer the 1:1 line indicating more accurate predictions. UL 5 and 6 are colored in purple and have triangle and square shape to distinguish their different AD types. As could be expected chemicals which are external to IFSQSAR have more uncertainty and variability in the predictions (RMSEP 1.00) compared to chemicals in the IFSQSAR validation dataset (RMSEP 0.57). The external data span a larger range of log *K*_*OW*_ values, from about −5 to 11, compared to the validation data which spans values from about −2 to 8. The chemicals in this expanded lower range tend to be flagged as out of the AD with UL 2 or UL 3 and are mostly identified as solids by the chemical state predictions. The chemicals in the middle of the range with over-predicted log *K*_*OW*_ values and which are mostly UL 2 and UL 3 are also mostly identified as solids and are mostly very large and complex chemicals.

Figure S2 shows the data for wet and dry *K*_*OW*_. Strong acids and bases and salts were not included because these data were likely distribution ratios (*D*_*OW*_) rather than *K*_*OW*_. Data in the IFSQSAR training dataset are excluded in these figures, only data in the IFSQSAR validation dataset and data that are in neither set are included. Applying the IFSQSAR model which applies the PPLFER equation for dry *K*_*OW*_ shows poorer statistics (RMSEP 1.19) compared to the model which applies the PPLFER equation for wet *K*_*OW*_ (RMSEP 0.98). As expected, the PPLFER for dry *K*_*OW*_ tends to underestimate the experimental *K*_*OW*_ values for more water-soluble chemicals, with the predictions skewing to lower values.

Figure S3 shows chemicals identified as liquids or solids plotted separately. Predicted *K*_*OW*_ values for liquids are more accurate with overall RMSEP 0.67 compared to 1.03 for solids. The ratio between RMSEP for solids and the RMSEP for liquids tends to increase with increasing RMSEP. More of the liquids are within the AD, 64% have aggregate UL 0 or 1 compared to solids which have only 31% assigned UL 0 or 1. This means that solids are more likely to be out of the AD, and regardless of whether they are in the AD or not, the *K*_*OW*_ predictions for solids are less accurate, though the difference is relative small for solids that are UL 0, 1, or 2. There are a few different reasons why the predictions for solids may be less accurate. Solids tend to be larger chemicals than liquids and fragment based QSPR predictions, such as the IFSQSAR solute descriptor QSPRs which are used in the PPLFER based models in this work, are known to be less accurate for larger chemicals [43, 52]. The functional group counts in larger chemicals are more likely to be outside of the range of values in the training dataset, meaning that the QSPRs must be extrapolated outside of their training set. Extrapolation is always more uncertain than interpolation between values within the range of the training dataset. Larger chemicals have more opportunities for intramolecular interactions between functional groups which can confound group contribution QSPRs such as those in IFSQSAR. Making experimental measurements for larger chemicals also tends to be more challenging because their solubility in some phases may be very low, so the experimental data also may be less accurate. For example, solids might be more likely to self-associate and undergo a phase transition at low concentrations in water or octanol such as has been observed for perfluorinated alkyl substances [53], which would have a confounding effect for interpreting experimental concentrations in octanol and water. Another example is polymorphism, where a chemical has multiple solid forms each with a different crystal structure and a different solubility [54]. This effect is well known in pharmaceutical science because it is an aspect of drug formulation but is not considered as much in environmental applications.

Table 3 summarizes statistics for the model evaluations and shows the fraction of each subset of data where the experimental values fall within the 95% PI calculated from the aggregate RMSEP estimates. For chemicals in the IFSQSAR validation dataset a little greater than 95% of the chemicals fall within in PI, which is to be expected because these chemicals are a subset of the data used to estimate the RMSEP. For the data which are external to IFSQSAR only 90% of chemicals fall within the 95% PI. The results are about the same for liquids vs. solids at 90% overall. The results are quite consistent across the different UL with no obvious trend. For liquids the fraction within the PI is more variable at UL 2 and UL 3 due to the small number of chemicals. Adjusted RMSEP estimates will be made for all QSPRs in “IFSQSAR Uncertainty Estimates” section.

**Table 3:** Validation statistics for log *K*_*OW*_*,* log *K*_*AW*_, and log *K*_*OA*_

	UL	log K_OW_			log K_AW_			log K_OA_		
		RMSEP	%in	n	RMSEP	%in	n	RMSEP	%in	n
All data (validation and external datasets) (Figure S2A, No Figure, No Figure)	All UL	0.98	91	9120	1.27	85	379	0.61	97	156
	UL 0	0.45	89	225	0.48	100	8	0.16	100	1
	UL 1	0.62	91	3134	1.10	81	215	0.33	96	76
	UL 2	0.97	91	5024	1.38	90	153	0.80	99	77
	UL 3	1.89	91	700	5.18	50	2	0.85	100	2
	UL 5	2.68	92	37	2.67	0	1			0
IFSQSAR validation dataset (Fig. 2A, Figure S4A, Figure S6A)	All UL	0.57	96	704	1.05	89	124	0.35	98	50
	UL 0	0.32	98	40	0.45	100	6	0.16	100	1
	UL 1	0.36	96	417	0.77	89	88	0.25	97	36
	UL 2	0.75	96	226	1.36	90	29	0.55	100	13
	UL 3	1.37	100	21	5.56	0	1			0
	UL 5			0	1.05	89	124			0
IFSQSAR external dataset (Fig. 2B, Figure S4B, Figure S6B)	All UL	1.00	90	8416	1.36	83	255	0.70	97	106
	UL 0	0.48	88	185	0.56	100	2			0
	UL 1	0.65	90	2717	1.28	76	127	0.39	95	40
	UL 2	0.98	90	4798	1.38	90	124	0.84	98	64
	UL 3	1.91	90	679	4.78	100	1	0.85	100	2
	UL 5	2.68	92	37	2.67	0	1			0
IFSQSAR external dataset liquids (Figure S3A, Figure S5A, No Figure)	All UL	0.67	90	785	0.92	90	42			0
	UL 0	0.42	90	20	0.37	100	1			0
	UL 1	0.55	91	486	0.97	91	22			0
	UL 2	0.83	88	266	0.64	94	18			0
	UL 3	1.17	75	8			0			0
	UL 5	1.69	100	5	2.67	0	1			0
IFSQSAR external dataset solids (Figure S3B, Figure S5B, see Figure S6B)	All UL	1.03	90	7631	1.43	81	213	0.70	97	106
	UL 0	0.48	87	165	0.70	100	1			0
	UL 1	0.67	90	2231	1.33	73	105	0.39	95	40
	UL 2	0.98	90	4532	1.47	89	106	0.84	98	64
	UL 3	1.91	90	671	4.78	100	1	0.85	100	2
	UL 5	2.81	91	32	1.43	81	213			0

There are much fewer data available for *K*_*AW*_ and *K*_*OA*_ than for *K*_*OW*_; therefore, the statistics are less reliable, but the results are consistent with the general trends observed for *K*_*OW*_. Figures analogous to Figs. 2A, B, and S3 are shown in the SI for *K*_*AW*_ and *K*_*OA*_ (Figure S4 through Figure S6). The prediction statistics are better for chemicals in the validation dataset than in the dataset of chemicals external to IFSQSAR as shown in Figure S4 for *K*_*AW*_ and Figure S6 for *K*_*OA*_. Figure S5 shows the prediction statistics for liquids are better than for solids for *K*_*AW*_, while for *K*_*OA*_ the external test set chemicals are all solids. Table 3 also summarizes the validation statistics for the performance of the *K*_*AW*_ and *K*_*OA*_ models. The fraction of experimental data which falls within the 95% PI is much more variable compared to the data for *K*_*OW*_, likely due the limited amount of data, but again the overall trend is similar, chemicals in the validation set are within the PI more than chemicals in the external set, and liquids are within the PI about as often as solids.

### Evaluation of IFSQSAR VP and S_W_ predictions

There are two IFSQSAR methods for predicting *VP* and *S*_*W*_; the indirect method developed in previous work [32, 34] and the direct method developed in the current study as described in Section SI-4. The indirect method predicts system parameters for *VP* and then calculates system parameters for *S*_*W*_ by thermodynamic property cycle, while the direct method uses system parameters calibrated with experimental data for *S*_*W*_ and uses a thermodynamic property cycle to calculate system parameters for *VP*. Table 4 shows the validation statistics for the *VP* and *S*_*W*_ direct method predictions, and Table S1 and Table S2 in the SI compare the direct predictions to the indirect predictions and direct predictions with the Van’t Hoff correction applied. Section SI-4 briefly describes theoretical reasons that *VP* and *S*_*W*_ will be different for liquids and solids. The indirect method was trained only on liquids and is not applicable to solid chemicals, the RMSEPs for predicting properties for solids, i.e., *VP*_*[s]*_ and *S*_*W[s]*_, are 5 to 6, respectively (results not shown). Figure S7 shows the indirect method gives good predictions for *VP*_*[l]*_ and *S*_*W[l]*_ with RMSEP values of 0.78 and 0.96, respectively, for chemicals which are external to IFSQSAR, i.e. are not in either the training or validation dataset of the IFSQSAR solute descriptor QSPRs.

**Table 4:** Validation statistics for log *VP* and log *S*_*W*_

	UL	log VP			log S_W_		
		RMSEP	%in	n	RMSEP	%in	n
All data (validation and external datasets)	All UL	1.51	80	1699	1.77	72	3338
	UL 0	0.73	81	58	1.34	73	92
	UL 1	0.89	88	866	1.50	69	1212
	UL 2	1.94	72	680	1.77	76	1440
	UL 3	3.11	69	45	2.51	88	117
	UL 5	3.14	82	11	1.95	88	8
	UL 6	0.95	56	39	2.23	66	469
IFSQSAR validation dataset	All UL	0.94	88	492	1.47	77	529
	UL 0	0.76	76	29	1.11	75	28
	UL 1	0.76	92	322	1.26	75	289
	UL 2	1.29	85	116	1.49	83	152
	UL 3	1.73	88	8	1.77	100	14
	UL 5			0			0
	UL 6	1.03	47	17	2.38	67	46
IFSQSAR external dataset (Figure S8B, Figure S9B)	All UL	1.69	77	1207	1.82	72	2809
	UL 0	0.70	86	29	1.43	72	64
	UL 1	0.96	86	544	1.57	67	923
	UL 2	2.05	69	564	1.80	76	1288
	UL 3	3.33	65	37	2.60	86	103
	UL 5	3.14	82	11	1.95	88	8
	UL 6	0.89	64	22	2.21	65	423
IFSQSAR external dataset liquids	All UL	0.71	90	495	0.88	91	464
	UL 0	0.62	86	21	0.62	94	16
	UL 1	0.59	91	308	0.76	90	232
	UL 2	0.88	92	134	1.01	93	182
	UL 3	1.43	67	3	1.06	100	4
	UL 5	1.06	100	7	0.95	67	3
	UL 6	0.89	64	22	1.01	93	27
IFSQSAR external dataset solids	All UL	2.11	68	712	1.96	68	2345
	UL 0	0.89	88	8	1.61	65	48
	UL 1	1.30	79	236	1.76	59	691
	UL 2	2.30	62	430	1.89	73	1106
	UL 3	3.45	65	34	2.64	86	99
	UL 5	5.01	50	4	2.36	100	5
	UL 6			0	2.27	64	396

The direct method predicts *VP* and S_W_ specifically for liquids and supercooled liquids if the chemical is a solid at 25 °C. When applying the direct method to solids the predictions need to be converted to *VP*_[s]_ and *S*_*W[s]*_ using the Van’t Hoff equation and Δ*S*_*M*_ and *T*_*M*_ which can be predicted by QSPRs in the IFSQSAR software. These additional QSPR predictions will introduce more uncertainty and variability into the predicted values for solids and the predictions would be expected to be less accurate. Because of this additional uncertainty the prediction accuracy of *VP*_*[s]*_ and *S*_*W[s]*_ using the Van’t Hoff correction is no better than just using the supercooled liquid predictions when comparing to the experimental data. Nevertheless, we present the results here for thoroughness because comparing the supercooled predictions to experimental *VP*_*[s]*_ and *S*_*W[s]*_ is an end-point mismatch. Large predictions for *VP* and *S*_*W*_ are capped to provide more reasonable values and assigned UL 6 corresponding to a boundary condition violation. Aside from the challenges for predicting properties for solids, much the same trends are observed in the data and model performance as observed for the partition ratios.

Figure S8 shows the predictions for *VP* using the direct method versus experimental values for chemicals external to IFSQSAR, comparing the effect of correcting with the Van’t Hoff equation or leaving the data uncorrected. Figure 2C, D show predictions corrected with the Van’t Hoff equation for data that are in the IFSQSAR validation dataset and data that are external to IFSQSAR. As is observed for the log *K* values, predictions for chemicals in the validation dataset (RMSEP 0.91) are more accurate than predictions for external chemicals (RMSEP 2.04). Predictions for liquids are again more accurate (RMSEP 0.71) than predictions for solids (RMSEP 2.59). Table 4 and Table S1 show the statistics for IFSQSAR log *VP* predictions. The trend is again the same for log *S*_*W*_, with predictions for chemicals in the validation dataset (RMSEP 1.28) more accurate than predictions for external chemicals (RMSEP 1.69) as shown in Fig. 2E, F, and predictions for liquids (RMSEP 0.88) more accurate than predictions for solids (RMSEP 1.81). Figure S9 shows the data with and without being corrected with the Van’t Hoff equation, and Table 4 and Table S2 show the statistics for IFSQSAR log *S*_*W*_ predictions.

The indirect and direct IFSQSAR methods for predicting log *VP*_*[l]*_ and log *S*_*W[l]*_ have comparable RMSEP and AD coverage; therefore, the direct method is preferable because the model has fewer inputs. For chemicals flagged as UL 0, 1, 2, 6 the IFSQSAR model predictions for solids with the Van’t Hoff correction applied have comparable or better RMSEP compared to the predictions with no correction applied. However, for chemicals flagged as being egregiously outside of the AD with UL 3 or UL 5 the IFSQSAR predictions with no Van’t Hoff correction applied have a better RMSEP. This can be interpreted to mean that if the IFSQSAR predictions are already very far outside of the AD adding further correction factors with their own AD and uncertainty is likely to only make the predictions worse.

### IFSQSAR uncertainty estimates

Tables 3 and 4 show the IFSQSAR 95% PIs typically capture about 80–90% of the deviations from experimental data for the external dataset, indicating a slight underestimation of the standard error of prediction. Multiplying the estimated RMSEP by 1.25 for all IFSQSAR QSPRs brought the fraction within the 95% PI of the partition ratio models close to 95%. No further adjustments were required for the partition ratio QSPRs. For the VP and *S*_*W*_ QSPRs there is a tendency for the 95% PIs to capture less than 95% of the predictions for solids; therefore, additional multiplicative adjustment factors of 1.67 and 1.25 were applied to the 95% PIs for the *VP* and *S*_*W*_ QSPRs respectively for chemicals identified as maybe or likely solids by the IFSQSAR state classifier. After these adjustments there was still a tendency for the *VP* QSPR to capture less than 95% for chemicals with high UL; therefore, an additional multiplicative adjustment factor of 1.25 is applied to chemicals with UL 2, UL 3, and UL 5.

The method proposed by Endo to calculate the prediction interval of PPLFER equations [35] was applied to see if the additional uncertainty of extrapolating outside of the PPLFER equation training dataset could explain why some chemicals were not within the estimated RMSEPs. As shown by Endo this is not a large source of additional uncertainty for PPLFERs with at least 100 chemicals in the training dataset, and all PPLFERs used in this work have hundreds of chemicals in their training datasets. The increase in RMSEP from applying this method rarely made any difference in the fraction of chemicals that were within the 95% PI.

## Conclusions

In summary, by applying the methods outlined in this study reasonable PIs can be assigned to all IFSQSAR PPLFER predictions for partition ratios, typically even those which are flagged as out of the AD and assigned UL 2 or UL 3. The main exceptions where a PI cannot be reasonably estimated are cases where the experimental endpoint is not applicable to the chemical in question, e.g., log *K*_*OW*_ at pH 7 of a strong acid. If a chemical is a valid target for the QSPR endpoint, then even if the prediction is out of AD, the model predictions are still useful when an acceptable level of uncertainty from the 95% PI estimation is determined. The acceptable level of uncertainty in a property prediction is fundamentally specific to an end user’s judgement and decision-context. For example, for priority setting or screening-level application of the IFSQSAR models, a higher level of uncertainty may be more tolerable than for a definitive risk assessment scenario. Given that typical experimental variability is about 0.1 log units for log *K*_*OW*_, and standard errors for PPLFERs with experimental solute descriptors are about 0.2 log units, a RMSEP of about 0.5 for chemicals within the AD of the models is probably an acceptable level of uncertainty for many decision-contexts. Even predictions which are out of the AD will typically have an RMSEP that gives a PI which is smaller than the full range of possible values for a partitioning property.

In general, the methods presented here predict partition ratios as log *K* for novel chemicals with an overall RMSEP of about 1 log unit. The RMSEP of log *K*_*AW*_ is a little larger and log *K*_*OA*_ is a little smaller than 1 log unit. This may have to do with the relative difficulties in making the measurements, or in making predictions for them. The log *K*_*AW*_ measurements have more experimental difficulties because of ionization and other effect specific to water so the inherent variability may be larger; however, there are fewer log *K*_*OA*_ measurements so the dataset of log *K*_*OA*_ values may not represent the full range of variability. *VP* and *S*_*W*_ of liquids are also predicted with an RMSEP of about one log unit, but predictions for solids have larger RMSEP, up to 2 log units or more depending on the subset. Many of these predictions are still good, for example 85% of predictions which are out of the AD for solid chemicals in the external *VP* dataset are within ± 1.98 log units of the expected value, corresponding to the 95% PI of an RMSEP of 1. The high overall RMSEP for *VP* and *S*_*W*_ of solids are clearly heavily influenced by a relatively small group of outliers. These instances tend to be strongly under-predicted, apparently due at least in part to the liquid to solid correction done with the Van’t Hoff equation. This disparity in prediction accuracy between liquids and solids is also apparent even for *K* values where it should theoretically not be an issue and warrants further investigation which will be a part of future work.

The new work described here advances the capacity for estimating uncertainty in PC property predictions, particularly for novel chemicals, and future work will show how these new methods and existing property predictions methods can be used to systematically address uncertainty in PC property data through integrated approaches to testing and assessment.

### Supplementary Information

Supplementary material 1.

## Data Availability

The data and model predictions included in this study are available in a user-friendly online platform, the Exposure And Safety Estimation (EAS-E) Suite (www.eas-e-suite.com). The IFSQSAR source code is available on github: https://github.com/tnbrowncontam/ifsqsar.

## Abbreviations

AD: Applicability domain
EAS-E (Suite): Exposure And Safety Estimation Suite
H: Henry’s Law constant
IFSQSAR: Python package for chemical property prediction used in this work
K: Partition ratio
K_AW_: Air–water partition ratio (K_AW_ = H/RT)
K_OA_: Octanol–air partition ratio (Henry’s Law constant for octanol)
K_OW_: Octanol–water partition ratio, mutually saturated (wet)
dry K_OW_: Octanol–water partition ratio, not mutually saturated (dry)
K_O[w]O[d]_ or K_OO_: Hypothetical partition ratio between wet and dry octanol
MV_[l]_ or MV: Molar volume of liquids or super-cooled liquids
MW: Molecular weight
OPERA: OPEn structure–activity/property Relationship App
PC (property): Physical–chemical property
PI: Prediction interval
PPLFER: Poly-parameter linear free energy relationship (Abraham solvation model)
R: Ideal gas law constant
RMSEP: Root mean squared error of prediction
SMILES: Simplified molecular input line entry system
S, A, B, V, L: PPLFER solute descriptors (Abraham descriptors)
s, a, b, v, l, c: PPLFER system parameters (Abraham equation system parameters)
S_A_: “Solubility” in air (S_A_ = VP/RT)
S_W[l]_ or S_W_: Solubility in water of liquids or super-cooled liquids
S_W[s]_: Solubility in water of solids
S_O[w][l]_: Solubility in wet octanol of liquids or super-cooled liquids
S_O[d][l]_ or S_O_: Solubility in dry octanol of liquids or super-cooled liquids
ΔS_M_: Entropy of melting
T: System temperature
T_B_: Boiling point
T_M_: Melting point
UL: Uncertainty Level
VP_[l]_ or VP: Vapor pressure of liquids or super-cooled liquids
VP_[s]_: Vapor pressure of solids
QSP(A)R: Quantitative structure–property (activity) relationship
UPPER: Unified physicochemical property estimation relationships
