# Supplementary material for: Identifying uncertainty in physical–chemical property estimation with IFSQSAR
Source: J Cheminform. 2024 May 30;16:65. doi: 10.1186/s13321-024-00853-w (PMC11140865; doi:10.1186/s13321-024-00853-w)
Supplement: Supplementary file 1 — Supplementary material 1. [file 13321_2024_853_MOESM1_ESM.docx]

**Supporting Information:** **Identifying Uncertainty in Physical-Chemical Property Estimation with IFSQSAR**

Trevor N. Brown^1*^, Alessandro Sangion^1^, Jon A. Arnot^1,2,3^

1. ARC Arnot Research & Consulting, Toronto, Ontario M4C 2B4, Canada

2. Department of Physical and Environmental Sciences, University of Toronto Scarborough, Toronto, Ontario M1C 1A4, Canada

3. Department of Pharmacology and Toxicology, University of Toronto, Toronto, Ontario M5S 1A8, Canada

* Corresponding author: Trevor N. Brown. trevor.n.brown@gmail.com

**SI Sections**

[SI-1: IFS-QSAR Details 2](#_Toc164071198)

[SI-2: New PPLFER for Predicting K_OW_ 2](#_Toc164071199)

[SI-3: New PPLFER for Predicting K_OO_ 3](#_Toc164071200)

[SI-4: New PPLFERs for Directly Predicting *S_A_*, *S_W_,* and *S_O_* 3](#_Toc164071201)

[SI-5: New Model for Predicting Physical State of Chemicals 5](#_Toc164071202)

[SI-6: New Model for Predicting Molar Volume of Liquids 6](#_Toc164071203)

[SI Figures 9](#_Toc164071204)

[SI-7: References 16](#_Toc164071205)

## SI-1: IFSQSAR Details

Iterative Fragment Selection (IFS) QSAR was developed and maintained by Trevor N. Brown and has been integrated into an online platform named EAS-E Suite for public use. The IFSQSAR development methods have been progressively updated and applied to other key chemical properties over the last 10 years [1-3]. They are fragment-based MLR models developed using extensive cross-validation and conservative goodness of fit metrics to create robust and predictive models, and make predictions based only the chemical structure as a SMILES string [4,5]. Development has been described in detail in the previous publications cited but will be described here in brief. IFSQSARs development adheres to the OECD 5 principles for development of QSARs for application in regulatory decision making [6,7]. These principles are 1: a defined endpoint; 2: an unambiguous algorithm; 3: a defined applicability domain (AD); 4: appropriate measures of predictivity; and 5: a mechanistic interpretation. The first step in any Quantitative Structure-(Activity)Property Relationships (QS(A)PRs) development is selecting an appropriate dataset and checking its accuracy, including the property values and chemical structures, to adhere with OECD principle 1.

The IFSQSAR development algorithm proceeds in five stages: descriptor pool generation, dataset splitting, descriptor pool filtering, model selection, and model validation. First a pool of fragments is generated from the chemicals in the dataset by breaking their structures into pieces, and the fragments are converted to SMARTS sub-structure search strings as implemented in the OpenBabel software package [8]. Several sub-pools of fragments are defined ranging from small, broadly applicable fragments to large, specific fragments. Being fragment based helps IFSQSARs adhere to OECD principles 2 and 5. Second the dataset is split into a training and validation dataset with an algorithm that ensures the two datasets include the broadest range of fragments as is possible. The training dataset is used to calibrate the QSPRs and the validation dataset is used to validate it only after the training is complete, to adhere to OECD principle 4. Third the pool of fragments is filtered to include only those present in the training dataset, and then further filtered to reduce the size of the descriptor pool because this reduces the possibility of chance correlations and overfitting. Fourth the QSPR model is created by selecting fragments from the pool and generating models by MLR of the property values vs. the fragment counts. Fragments are selected in iterative rounds from each sub-pool of fragments by forwards addition, replacement, and backwards elimination. Cross-validation is applied, and fragment selection is based on goodness-of-fit metrics calculated from the predictive sum of squares to ensure models with good predictive power. This simple and robust algorithm ensures adherence to OECD principle 2.

Calculating aggregate UL is straight forward for UL 0 to 3. For UL 4 some solute descriptors (E, S, A, B) are treated as UL 1 and for the L solute descriptor UL 4 is treated as UL 2 when calculating the aggregate. The A and B solute descriptors may have UL 6 if the QSPR predicted negative values, these are treated as UL 3 for calculating the aggregate UL. UL 5 always overrides any other UL from other inputs and sets the aggregate UL to UL 5.

## SI-2: New PPLFER for Predicting K_OW_

Data for log K_OW_ were taken from the paper in which the Poly-Parameter Free Linear Energy Relationship (PPLFER) equation was originally calibrated.[9] The solute descriptors were updated with more recent reliable values where available from previous research.[10,11] The PPLFER was then recalibrated in the form of **Equation 1** of the main text, and the new system parameters are provided in **Table 1** of the main text. See the original publications for the data used for this recalibration.

## SI-3: New PPLFER for Predicting K_OO_

The K_OO_ or K_O[w]O[d]_ is the hypothetical partition ratio between wet octanol (octanol saturated with water) and dry octanol (pure octanol). See **Section 2.1** of the main text for more discussion on how this difference arises from experimental data. The system parameters for K_OO_ were derived by subtracting the system parameters for dry log K_OW_ from those of wet log K_OW_. This implicitly attributes all of the discrepancy between the two log K_OW_ equations to the difference between wet and dry octanol, but there will also be some amount of experimental variability in the equations which also be included in the derived log K_OO_ system parameters. The dry K_OW_ is also hypothetical and calculated by thermodynamic cycle from K_OA_ and K_AW_, so the aggregate uncertainty of the K_OO_ system parameters includes the uncertainty of three other PPLFER equations, which is reflected by its higher fitting error shown in **Table 1** of the main text. Because the system parameters are small this means the relative error will be high. However, the system parameters make mechanistic sense. The positive values for s, a, and b mean that polar and hydrogen bonding chemicals will partition more into the octanol containing some water. The v system parameter is negative, this system parameter is correlated with the energy required for a solute to form a cavity in the solvent and in water this energy requirement is high, so chemicals with large volumes will partition more to the octanol without water. This mechanistic sensibility gives confidence in the PPLFER equation despite the relative uncertainty due to its derivation.

## SI-4: New PPLFERs for Directly Predicting *S_A_*, *S_W_,* and *S_O_*

VP and S_W_ data from OPERA datasets that overlap with the training dataset of the IFSQSAR solute descriptor QSPRs were used to train the new PPLFER equations. The new PPLFER equations were then validated using the data from the OPERA datasets that overlap with the validation dataset of the IFSQSAR solute descriptor QSPRs. Matching chemicals between the datasets was done by comparing CAS numbers. Only data measured at temperatures in the range of 20-25 °C were used.

Experimental melting points (T_M_), and where necessary boiling points (T_B_), were collected for all chemicals in the training and validation datasets. These were collected first from the OPERA T_M_ and T_B_ datasets, then manually searched for in PubChem, and finally searched for in the product information sheets of reputable chemical suppliers. These values were used to definitively classify the chemicals as gases, liquids, or solids.

All gases were excluded from the dataset because the meaning of VP and S_W_ for gases differs from liquids and solids, and the values are above the proposed caps discussed in the main text. All liquids were included in the training and validation datasets. Solids were only included if experimental values for entropy of fusion (*ΔS_M_*) were available from previous work [3]. VP and S_W_ for included solids were then converted to supercooled values using the Van’t Hoff equation. The Hildebrand equation was also tested but resulted in poorer statistics when fitting the PPLFER equations.

All VP data were converted to S_W_ by first converting to S_A_ at the measurement temperature and then applying the three-solubility approach with log K_AW_ predicted from the PPLFER equation calibrated in previous work [11] and shown in **Table 1** of the main text. The PPLFER equation for S_W_ in the form of **Equation 3** of the main text, which is shown in **Table 1** of the main text, was fitted with the reliable solute descriptors compiled in previous work vs. the liquid and supercooled liquid S_W_ corrected with the Van’t Hoff equation for solids. The PPLFER equations for VP and S_O_ were then calculated by applying a thermodynamic cycle with the PPLFER system parameters of log K_AW_, and wet or dry log K_OW_ respectively.

A total of 732 data points were used to train the PPLFER equation for S_W_ and 385 data points were used for external validation. The RMSEP for all data points in the validation dataset was 0.65. For validation data points derived from VP data the RMSEP was 0.62, lower than the value for data points derived from S_W_ with an RMSEP of 0.69. As shown in **Table 1** of the main text the equivalent values for the training data are closer at 0.59 and 0.60, respectively. Liquids are also predicted better than solids in the validation data with RMSEP of 0.43 and 0.85, respectively. This is comparable to the fitting errors for liquids and solids in the training dataset. As described above this equation includes liquids and super-cooled solids in the training data and applies to both. As a test two PPLFERs were calibrated separately instead, one using only data for liquids and the other using only data for solids. These two equations were then applied to the liquids and solids in the validation dataset, but this did not appreciably improve the overall predictive power relative to the equation which applies to both liquids and super-cooled solids which is shown in **Table 1** of the main text. With the separate equations for liquids and solids the RMSEP for liquids was still close to 0.4 and for solids the RMSEP was still close to 0.8.

The PPLFER system parameters for wet S_O_ and dry S_O_ were calculated by thermodynamic cycle. Wet S_O_ is calculated by adding S_W_ and wet log K_OW_, and dry log S_O_ is calculated by adding log S_A_ and log K_OA_. The difference between to system parameters exactly matches the log K_OO_ system parameters. S_O_ values for 183 chemicals which overlap with the other datasets in this work were extracted from Admire and Yalkowsky [12], of which 112 were in the training dataset used to create the solute descriptor QSPRs. This dataset was judged to be too small and not sufficiently diverse for training a PPLFER equation for S_O_, but the data can act as an additional external validation. As is done for VP and S_W_, the IFSQSAR predictions for S_O_ are for liquids or super-cooled liquids, these predictions for the 74 chemicals that are not in the IFSQSAR solute descriptor QSPR training dataset are shown in **Figure S1A**. Correcting with Van’t Hoff equation using IFSQSAR predicted ΔS_M_ and T_M_ is shown in **Figure S1B**. It can be seen that the liquid S_O_ values vary over a limited range of values, less than three orders of magnitude, and many the values are capped at the inverse of MV. The corrected data vary over a larger range, though still a much smaller range than VP or S_W_ and match the experimental data much better than the uncorrected data. The corrected data have an overall RMSEP of 0.88, which is less than the RMSEP of VP or S_W_, though the dataset is small and likely not representative of the potential variability. The correction for solids pulls all the UL 6 chemicals below the MV cap and these are reclassified into their original UL.

**Figure S1.** Plots of IFSQSAR predictions vs. experimental S_O_ values (A) uncorrected S_O[liq]_ and (B) S_O[liq]_ with solids corrected with the Van’t Hoff equation


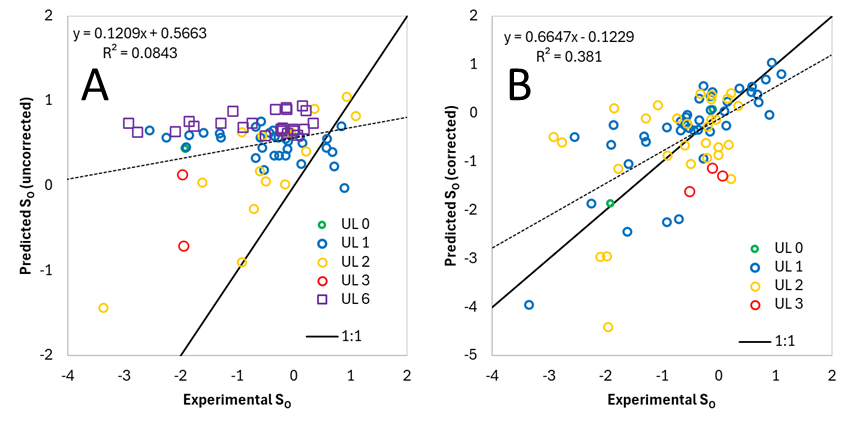


## SI-5: New Model for Predicting Physical State of Chemicals

The same dataset of chemicals used to train and validate the liquid MV QSPR (described in the next section) is used to train and validate the physical state classifier, but filtered so that only chemicals definitively classified by experimental *T_M_* and *T_B_* are included. The inputs for the physical state classifier are the IFSQSAR predicted solute descriptors, *T_M_* and *T_B_*. IFSQSAR has Meta QSPRs implemented to predict T_M_ and T_B_ based on PPLFER equations calibrated in previous work [11]. For T_M_ a consensus value with a previously created QSPR is used [3] calculated as the average of the two values. The solute descriptors are used to calculate the leverage of the chemical with regards to the liquid solutes identified in the IFSQSAR experimental solute descriptor training dataset. A higher leverage indicates the chemical is dissimilar to liquid chemicals, and this helps to distinguish between liquids and solids. Gases have solute descriptors that are not as different from liquids as the solids are so this method is not effective for distinguishing gases. Leverage cutoffs are defined the same way as is done in the IFSQSAR AD domain testing and UL_[liq]_ are assigned. Chemicals with UL_[liq]_ 0 or UL_[liq]_ 1 are likely to be liquids, and UL_[liq]_ 2 and UL_[liq]_ 3 are likely to be solids. The *T_M_* and *T_B_* are compared to the prediction temperature of 25 °C as another line of evidence to determine physical state, and a classification is made based on the decisions below. The *T_M_* and *T_B_* are uncertain predictions with relatively large RMSEP and prediction intervals (PI), *T_M_* more so than *T_B_*. Various PI were tested until the most accurate classification was achieved. Whichever of these decisions tests true first is the physical state classification.

1. [*T_B_* + 0.672∙RMSEP *T_B_* < 298.15K] = **likely gas**
2. [*T_M_ - 1.036*∙RMSEP *T_M_* > 298.15K *and* UL[liq] > 1] = **likely solid**
3. [*T_M_ + 1.96*∙RMSEP *T_M_* < 298.15K < *T_B_ - 1.96*∙RMSEP *T_B_* *and* UL[liq] <= 1] = **likely liquid**
4. [*T_M_* < 298.15K < *T_B_ and* UL[liq] <= 1] = **maybe liquid**
5. [*T_M_ + 1.96*∙RMSEP *T_M_* < 298.15K < *T_B_ - 1.96*∙RMSEP *T_B_* *and* UL[liq] > 1] = **maybe liquid**
6. [*T_M_* > 298.15K] = **maybe solid**
7. [*T_B_ <* 298.15K] = **maybe gas**
8. [*T_M_* < 225K *or* *T_B_ <* 425K] = **maybe liquid**
9. **maybe solid**

The accuracy of the physical state classification was assessed against chemicals in the validation dataset. As can be seen below the accuracy of the “likely” qualified classifications is high, greater than 95%, and the accuracy of the “maybe” qualified classifications is moderate, about 75-80%.

| classification | n | %correct |
| --- | --- | --- |
| maybe gas | 14 | 78.6 |
| **likely gas** | **4** | **100.0** |
| maybe liquid | 994 | 74.7 |
| **likely liquid** | **389** | **96.7** |
| maybe solid | 1240 | 80.6 |
| **likely solid** | **785** | **99.7** |

## SI-6: New Model for Predicting Molar Volume of Liquids

Molar Volume (MV) was calculated from density and Molecular Weight (MW) as MV (cm^3^/mole) = MW (g/mole) / density (g/cm^3^). Density data were collected from two sources. Previous work collected densities from PubChem (<https://pubchem.ncbi.nlm.nih.gov/>) to calculate MV for about 2000 chemicals in the training and validation datasets of the PPLFER solute descriptors which overlapped with the OPERA VP and S_W_ datasets [11]. Added to this was a large dataset of density values from OChem (<https://ochem.eu/>). This dataset contained about 60k datapoints; after merging duplicates, filtering out anomalous values, and removing inorganic chemicals, densities for about 13000 chemicals were added to the dataset. The smaller dataset collected from PubChem includes the temperature at which the density was measured, but this information is not included in OChem and is a credible source of uncertainty in the calculations.

The physical state, i.e. gas, liquid or solid, of each chemical at the density measurement temperature was classified by finding overlap with the OPERA T_M_ and T_B_ datasets wherever possible. The rest of the chemicals were classified using the IFSQSAR physical state classifier described in the previous section. Using this classification and density measurement temperature the dataset of densities collected from PubChem was assigned a density measurement type. The possible measurement types were:

1. Density of a liquid measured at ca. 25 °C (20-30 °C)
2. Density of a solid measured at ca. 25 °C (20-30 °C)
3. Density of a gas measured as a liquid at its boiling point
4. Density of a solid measured as a liquid at its melting point
5. Density of a gas at high pressure
6. Unclassified – insufficient data

For the OChem dataset the type of density measurement was assigned based on an assumed measurement temperature of 25 °C, and the IFSQSAR physical state classification. The possible measurement types were:

1. Density of a liquid measured at ca. 25 °C – when IFSQSAR assigned a “likely liquid” designation
2. Density of a solid measured at ca. 25 °C – when IFSQSAR assigned a “likely solid” designation
3. Unclassified – insufficient data

For training and validating the liquid MV QSPR only data points of measurement type 1, 3 and 4 of the PubChem dataset and measurement type 1 of the OChem dataset, i.e., density of a liquid measured at ca. 25 °C (20-30 °C), were considered. A small number of measurement type 3 data (16) and measurement type 4 data (30) were included in the training dataset to help expand the applicability domain of the QSPR. Chemicals in the PubChem dataset were assigned to the training or validation dataset based on whether the chemicals were in the training or validation dataset of the solute descriptor QSPRs [13]. The remaining values were sorted by density and MV and assigned alternately to the training and validation datasets.

All densities were converted to MV in units of cm^3^/mole for training the QSPR. There are 2094 chemicals in the training dataset and 2244 chemicals in the validation dataset. The descriptors used as inputs for the liquid MV QSPR are based mostly on a similar model by McGowan for the V solute descriptor [14]. The approach was to keep the model simple and robust, adding as few model descriptors as possible to achieve a good fit of the data. The model descriptors and the regression coefficients are shown here:

| Descriptor | Coefficient | Coef. Err. |
| --- | --- | --- |
| boron atom | 33.8142 | 1.2296 |
| carbon atom | 23.1256 | 1.0273 |
| nitrogen atom | 19.2123 | 0.8653 |
| oxygen atom | 20.1725 | 0.6031 |
| fluorine atom | 24.6036 | 0.4196 |
| silicon atom | 43.1347 | 1.0919 |
| phosphorous atom | 31.6932 | 1.7293 |
| sulfur atom | 30.6460 | 0.7136 |
| chlorine atom | 31.7958 | 0.3926 |
| germanium atom | 40.8333 | 1.3888 |
| arsenic atom | 42.9797 | 1.8276 |
| selenium atom | 34.9946 | 1.2584 |
| bromine atom | 35.3406 | 0.4776 |
| iodine atom | 41.6060 | 0.8173 |
| triple bond between non-hydrogen atoms | -10.8407 | 1.5968 |
| double bond between non-hydrogen atoms | -12.2985 | 1.0283 |
| single bond between non-hydrogen atoms | -14.8916 | 0.5978 |
| aromatic bond between non-hydrogen atoms | -14.0663 | 0.8024 |
| hydrogen atom | 4.3235 | 0.2488 |
| smallest set of smallest rings | 6.7080 | 0.4093 |

The fit of the model is very good with and R^2^ of 0.9991 and an RMSE of 4.75. The validation statistics are also very good, with RMSEP of 4.63, comparable to the training dataset RMSE and small compared to the range of values in the validation dataset (40.24 – 591.6 cm^3^/mole). Applying the QSPR to all chemicals not in the training dataset (about 13000 chemicals) gives an RMSEP of 11.34. The largest source of error is end-point mismatches; the largest RMSEP for a subset of chemicals is 14.68 for chemicals where the density data is identified as the density of a solid at ca. 25 °C, i.e. class 2 of the PubChem and OChem datasets above.

A conversion factor was fitted in this work to convert liquid MV to solid MV by minimizing the prediction errors for solids: MV_[s]_ = MV_[l]_ * 0.962. Applying this correction reduced the RMSEP for solids from 14.68 to 12.64. Refitting the same QSPR model to solids did not appreciably improve the fit, there are more complex interactions in the solid phase that the simplistic model calibrated here does not capture. However, the QSPRs developed here are intended to be used as inputs for mass balance models, and in this context, chemicals are typically assumed to be in their liquid or supercooled liquid forms, so this is not viewed as a limitation.

## SI Figures

**Figure S2.** Predicted vs. Experimental log *K_OW_* of all validation and external data for (A) wet log *K_OW_* and (B) dry log *K_OW_*


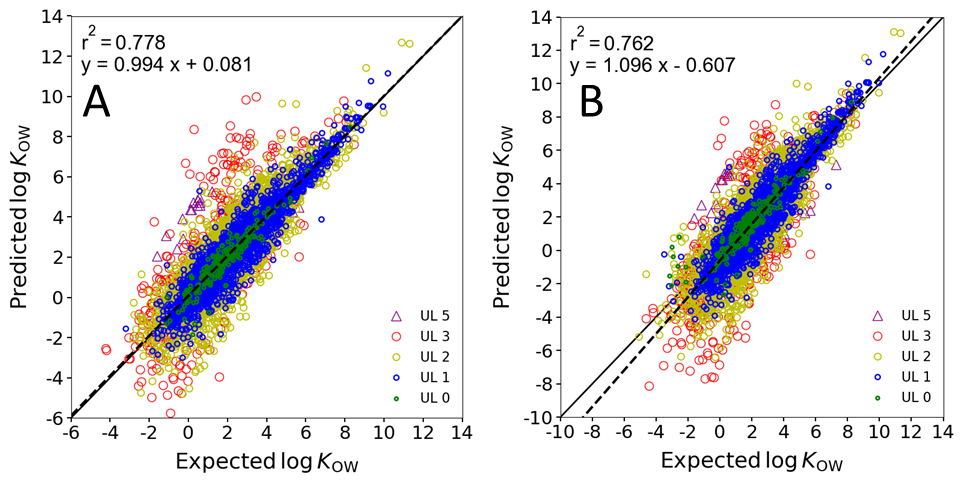


**Figure S3.** log *K_OW_* Predicted vs. Experimental of the external set for (A) liquids or gases and (B) solids


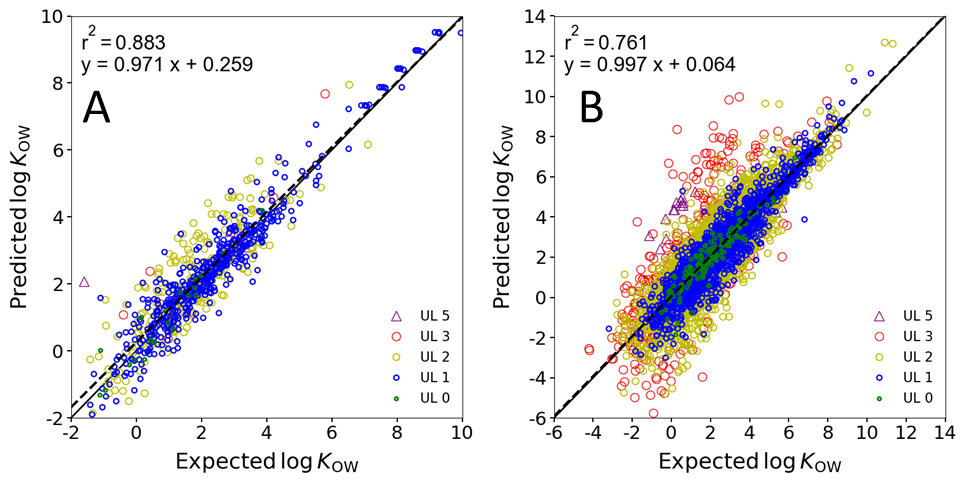


**Figure S4.** log K_AW_ Predicted vs. Experimental for (A) IFSQSAR validation Set and (B) external set


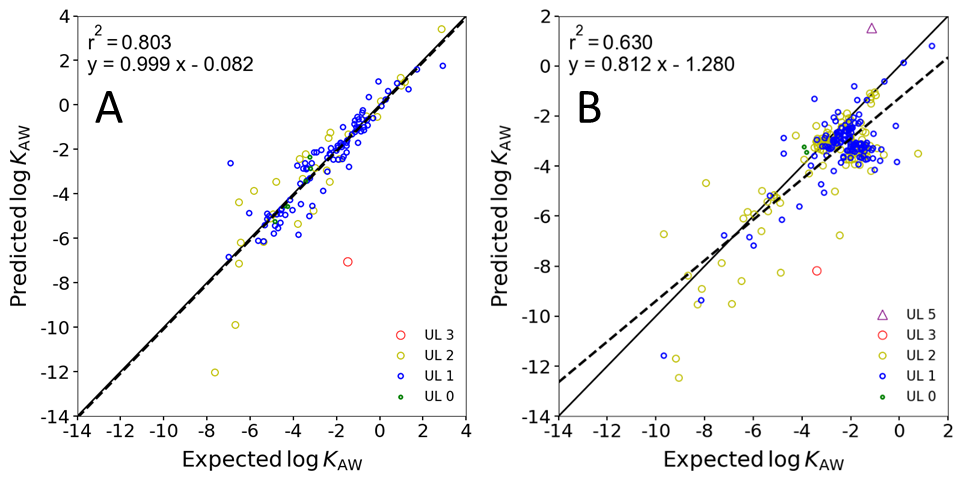


**Figure S5.** log K_AW_ Predicted vs. Experimental of the External Set for (A) Liquids or Gases and (B) Solids


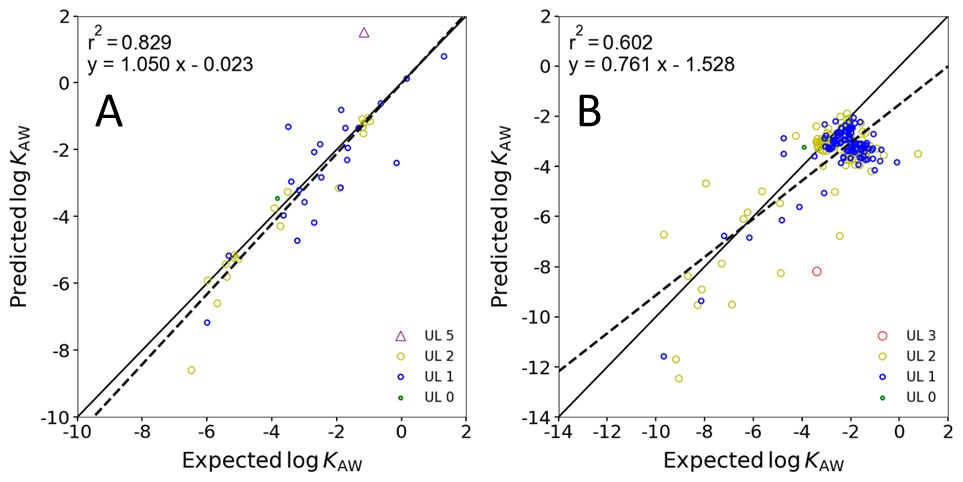


**Figure S6.** log K_OA_ Predicted vs. Experimental for (A) IFSQSAR validation set and (B) external set


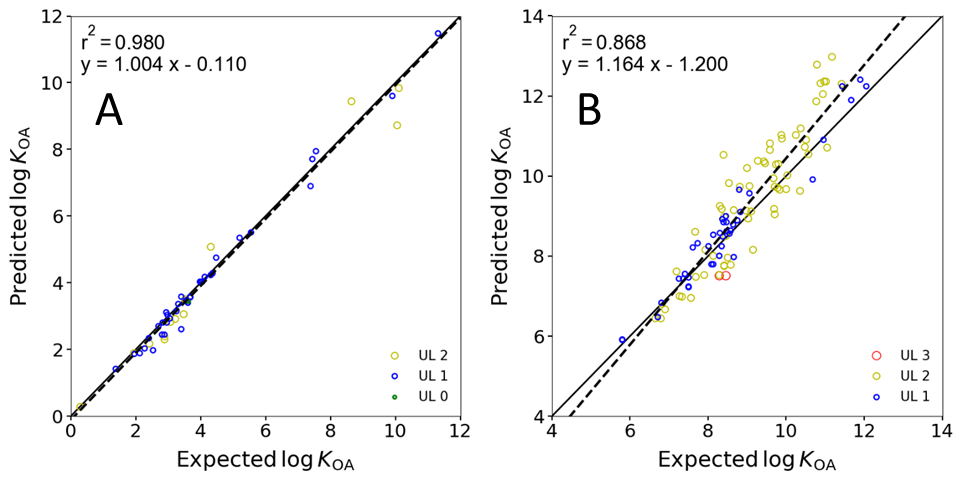


**Figure S7.** IFSQSAR Indirect Method vs. Experimental for (A) log *VP* and (B) log *S_W_* of liquids in the external set


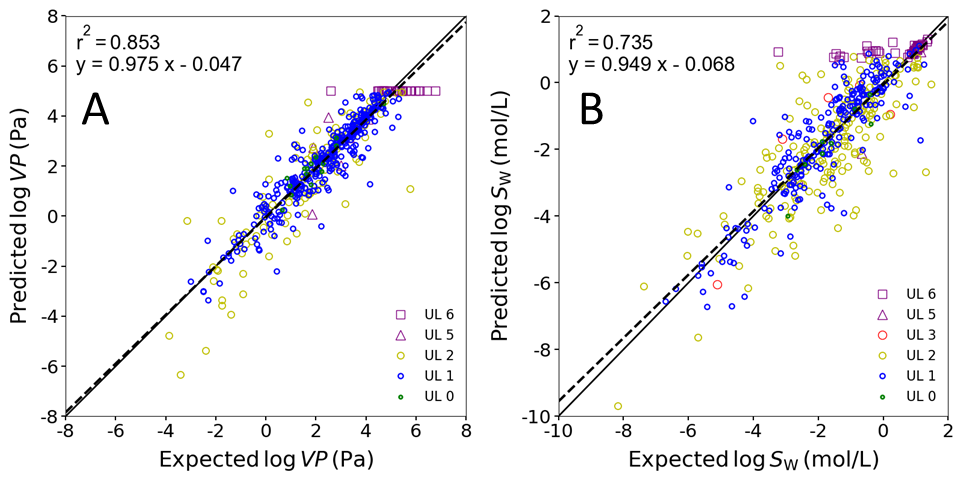


**Figure S8.** IFSQSAR Predicted vs. External Set for log VP which is (A) corrected with Van’t Hoff equation (same as **Figure 2D** for comparison) and (B) uncorrected


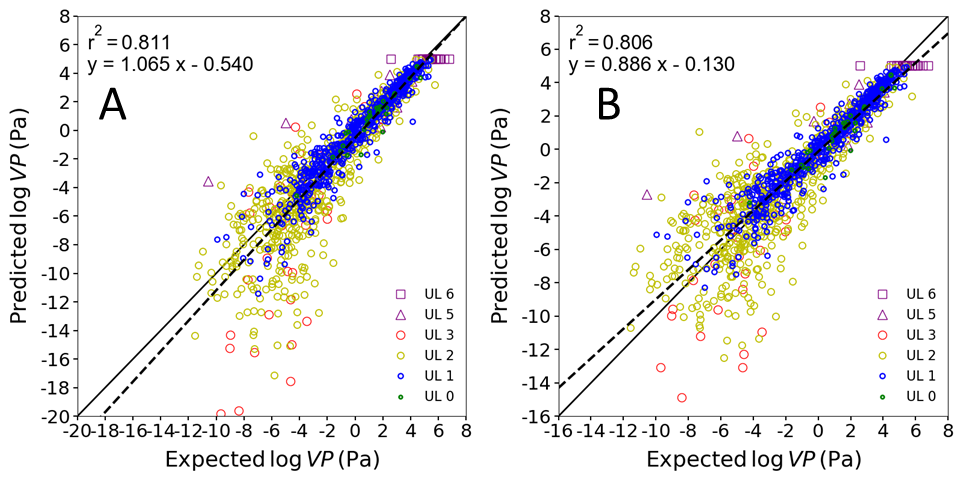


**Figure S9.** IFSQSAR Predicted vs. External Set for log S_W_ which is (A) corrected with Van’t Hoff equation (same as **Figure 2F** for comparison) and (B) uncorrected


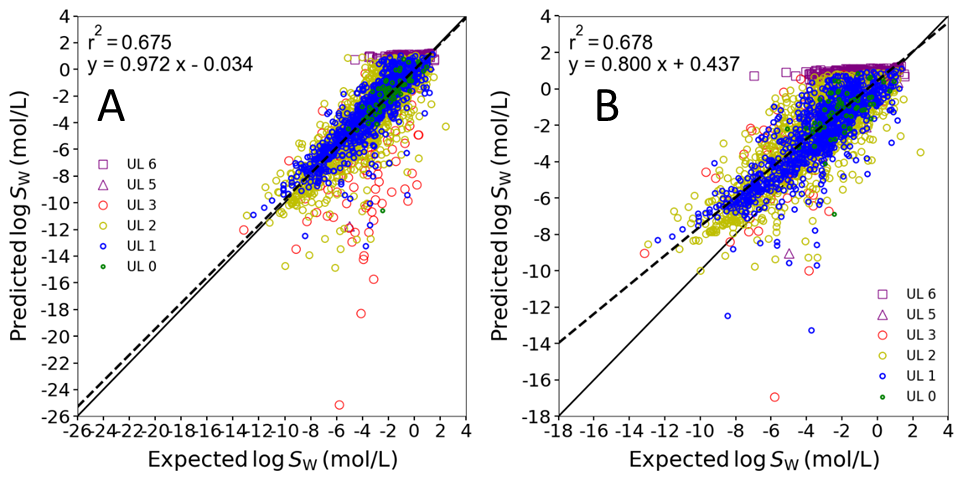


**Table S1. Validation Statistics for log VP**

|  |  | Indirect (liquids only) | | | | Direct (Van’t Hoff) | | | Direct (No correction) | | |
| --- | --- | --- | --- | --- | --- | --- | --- | --- | --- | --- | --- |
|  | UL | RMSEP | %in | n | RMSEP | | %in | n | RMSEP | %in | n |
| all data  (validation and external datasets) | all UL | 0.67 | 96 | 812 | 1.79 | | 80 | 1699 | 1.51 | 80 | 1699 |
|  | UL 0 | 0.47 | 96 | 26 | 0.75 | | 83 | 58 | 0.73 | 81 | 58 |
|  | UL 1 | 0.53 | 98 | 578 | 0.87 | | 88 | 866 | 0.89 | 88 | 866 |
|  | UL 2 | 1.02 | 93 | 152 | 2.29 | | 72 | 680 | 1.94 | 72 | 680 |
|  | UL 3 |  |  | 0 | 4.82 | | 62 | 45 | 3.11 | 69 | 45 |
|  | UL 5 | 1.00 | 100 | 7 | 2.88 | | 82 | 11 | 3.14 | 82 | 11 |
|  | UL 6 | 0.87 | 86 | 49 | 0.95 | | 56 | 39 | 0.95 | 56 | 39 |
| IFSQSAR validation dataset  (**No Figure**,  **Figure 2C**,  **No Figure**) | all UL | 0.47 | 97 | 317 | 0.91 | | 88 | 492 | 0.94 | 88 | 492 |
|  | UL 0 | 0.59 | 91 | 11 | 0.79 | | 83 | 29 | 0.76 | 76 | 29 |
|  | UL 1 | 0.36 | 100 | 248 | 0.66 | | 93 | 322 | 0.76 | 92 | 322 |
|  | UL 2 | 0.63 | 97 | 39 | 1.27 | | 84 | 116 | 1.29 | 85 | 116 |
|  | UL 3 |  |  | 0 | 2.33 | | 88 | 8 | 1.73 | 88 | 8 |
|  | UL 5 |  |  | 0 |  | |  | 0 |  |  | 0 |
|  | UL 6 | 0.98 | 74 | 19 | 1.03 | | 47 | 17 | 1.03 | 47 | 17 |
| IFSQSAR  external dataset  (**Figure S7A**,  **Figure 2D,**  **Figure S8B**) | all UL | 0.78 | 95 | 495 | 2.04 | | 77 | 1207 | 1.69 | 77 | 1207 |
|  | UL 0 | 0.35 | 100 | 15 | 0.70 | | 83 | 29 | 0.70 | 86 | 29 |
|  | UL 1 | 0.62 | 96 | 330 | 0.97 | | 85 | 544 | 0.96 | 86 | 544 |
|  | UL 2 | 1.12 | 92 | 113 | 2.45 | | 70 | 564 | 2.05 | 69 | 564 |
|  | UL 3 |  |  | 0 | 5.20 | | 57 | 37 | 3.33 | 65 | 37 |
|  | UL 5 | 1.00 | 100 | 7 | 2.88 | | 82 | 11 | 3.14 | 82 | 11 |
|  | UL 6 | 0.79 | 93 | 30 | 0.89 | | 64 | 22 | 0.89 | 64 | 22 |
| IFSQSAR  external dataset  liquids | all UL | 0.78 | 95 | 495 | 0.71 | | 89 | 495 | 0.71 | 90 | 495 |
|  | UL 0 | 0.35 | 100 | 15 | 0.62 | | 86 | 21 | 0.62 | 86 | 21 |
|  | UL 1 | 0.62 | 96 | 330 | 0.59 | | 91 | 308 | 0.59 | 91 | 308 |
|  | UL 2 | 1.12 | 92 | 113 | 0.88 | | 92 | 134 | 0.88 | 92 | 134 |
|  | UL 3 |  |  | 0 | 1.43 | | 67 | 3 | 1.43 | 67 | 3 |
|  | UL 5 | 1.00 | 100 | 7 | 1.06 | | 100 | 7 | 1.06 | 100 | 7 |
|  | UL 6 | 0.79 | 93 | 30 | 0.89 | | 64 | 22 | 0.89 | 64 | 22 |
| IFSQSAR  external dataset  solids | all UL |  |  |  | 2.59 | | 67 | 712 | 2.11 | 68 | 712 |
|  | UL 0 |  |  |  | 0.89 | | 75 | 8 | 0.89 | 88 | 8 |
|  | UL 1 |  |  |  | 1.31 | | 78 | 236 | 1.30 | 79 | 236 |
|  | UL 2 |  |  |  | 2.76 | | 63 | 430 | 2.30 | 62 | 430 |
|  | UL 3 |  |  |  | 5.41 | | 56 | 34 | 3.45 | 65 | 34 |
|  | UL 5 |  |  |  | 4.56 | | 50 | 4 | 5.01 | 50 | 4 |
|  | UL 6 |  |  |  |  | |  | 0 |  |  | 0 |

**Table S2. Validation Statistics for** **log S_W_**

|  |  | Indirect (liquids only) | | | | Direct (Van’t Hoff) | | | Direct (No correction) | | |
| --- | --- | --- | --- | --- | --- | --- | --- | --- | --- | --- | --- |
|  | UL | RMSEP | %in | n | RMSEP | | %in | n | RMSEP | %in | n |
| all data  (validation and external datasets) | all UL | 0.86 | 96 | 703 | 1.63 | | 88 | 3338 | 1.77 | 72 | 3338 |
|  | UL 0 | 0.43 | 100 | 16 | 1.23 | | 90 | 99 | 1.34 | 73 | 92 |
|  | UL 1 | 0.70 | 97 | 379 | 1.17 | | 87 | 1262 | 1.50 | 69 | 1212 |
|  | UL 2 | 1.04 | 96 | 241 | 1.52 | | 89 | 1608 | 1.77 | 76 | 1440 |
|  | UL 3 | 1.03 | 100 | 6 | 4.16 | | 88 | 161 | 2.51 | 88 | 117 |
|  | UL 5 | 0.87 | 100 | 3 | 2.56 | | 78 | 9 | 1.95 | 88 | 8 |
|  | UL 6 | 1.00 | 95 | 58 | 1.55 | | 85 | 199 | 2.23 | 66 | 469 |
| IFSQSAR validation dataset  (**No Figure**,  **Figure 2E**,  **No Figure**) | all UL | 0.62 | 97 | 239 | 1.28 | | 88 | 529 | 1.47 | 77 | 529 |
|  | UL 0 | 0.36 | 100 | 7 | 1.29 | | 81 | 31 | 1.11 | 75 | 28 |
|  | UL 1 | 0.58 | 98 | 163 | 0.98 | | 86 | 293 | 1.26 | 75 | 289 |
|  | UL 2 | 0.79 | 96 | 54 | 1.22 | | 92 | 165 | 1.49 | 83 | 152 |
|  | UL 3 | 0.29 | 100 | 1 | 3.79 | | 89 | 19 | 1.77 | 100 | 14 |
|  | UL 5 |  |  | 0 |  | |  | 0 |  |  | 0 |
|  | UL 6 | 0.40 | 100 | 14 | 0.91 | | 95 | 21 | 2.38 | 67 | 46 |
| IFSQSAR  external dataset  (**Figure S7B**,  **Figure 2F,**  **Figure S9B**) | all UL | 0.96 | 96 | 464 | 1.69 | | 88 | 2809 | 1.82 | 72 | 2809 |
|  | UL 0 | 0.47 | 100 | 9 | 1.21 | | 94 | 68 | 1.43 | 72 | 64 |
|  | UL 1 | 0.78 | 96 | 216 | 1.22 | | 87 | 969 | 1.57 | 67 | 923 |
|  | UL 2 | 1.10 | 96 | 187 | 1.55 | | 89 | 1443 | 1.80 | 76 | 1288 |
|  | UL 3 | 1.12 | 100 | 5 | 4.21 | | 88 | 142 | 2.60 | 86 | 103 |
|  | UL 5 | 0.87 | 100 | 3 | 2.56 | | 78 | 9 | 1.95 | 88 | 8 |
|  | UL 6 | 1.12 | 93 | 44 | 1.61 | | 84 | 178 | 2.21 | 65 | 423 |
| IFSQSAR  external dataset  liquids | all UL | 0.96 | 96 | 464 | 0.88 | | 91 | 464 | 0.88 | 91 | 464 |
|  | UL 0 | 0.47 | 100 | 9 | 0.62 | | 94 | 16 | 0.62 | 94 | 16 |
|  | UL 1 | 0.78 | 96 | 216 | 0.76 | | 90 | 232 | 0.76 | 90 | 232 |
|  | UL 2 | 1.10 | 96 | 187 | 1.01 | | 93 | 182 | 1.01 | 93 | 182 |
|  | UL 3 | 1.12 | 100 | 5 | 1.06 | | 100 | 4 | 1.06 | 100 | 4 |
|  | UL 5 | 0.87 | 100 | 3 | 0.95 | | 67 | 3 | 0.95 | 67 | 3 |
|  | UL 6 | 1.12 | 93 | 44 | 1.01 | | 93 | 27 | 1.01 | 93 | 27 |
| IFSQSAR  external dataset  solids | all UL |  |  |  | 1.81 | | 87 | 2345 | 1.96 | 68 | 2345 |
|  | UL 0 |  |  |  | 1.34 | | 94 | 52 | 1.61 | 65 | 48 |
|  | UL 1 |  |  |  | 1.33 | | 86 | 737 | 1.76 | 59 | 691 |
|  | UL 2 |  |  |  | 1.61 | | 88 | 1261 | 1.89 | 73 | 1106 |
|  | UL 3 |  |  |  | 4.27 | | 88 | 138 | 2.64 | 86 | 99 |
|  | UL 5 |  |  |  | 3.06 | | 83 | 6 | 2.36 | 100 | 5 |
|  | UL 6 |  |  |  | 1.69 | | 83 | 151 | 2.27 | 64 | 396 |

## SI-7: References

1. Brown TN, Arnot JA, Wania F (2012) Iterative fragment selection: A group contribution approach to predicting fish biotransformation half-lives. Environmental Science and Technology 46:8253-8260. doi:10.1021/es301182a

2. Arnot JA, Brown TN, Wania F (2014) Estimating screening-level organic chemical half-lives in humans. Environmental Science and Technology 48:723-730. doi:10.1021/es4029414

3. Brown TN, Armitage JM, Arnot JA (2019) Application of an Iterative Fragment Selection (IFS) Method to Estimate Entropies of Fusion and Melting Points of Organic Chemicals. Molecular Informatics 38 (8-9):1800160. doi:10.1002/minf.201800160

4. Weininger D (1988) SMILES, a chemical language and information system. 1. Introduction to methodology and encoding rules. J Chem Inf Comp Sci 28 (1):31-36. doi:10.1021/ci00057a005

5. Weininger D, Weininger A, Weininger JL (1989) SMILES. 2. Algorithm for generation of unique SMILES notation. J Chem Inf Comp Sci 29 (2):97-101. doi:10.1021/ci00062a008

6. OECD (2004) OECD Principles for the Validation, for Regulatory Purposes, of (Quantitative) Structure-Activity Relationship Models. OECD, Paris

7. OECD (2007) Guidance Document on the Validation of (Quantitative) Structure-Activity Relationships (QSAR) Models. OECD Environment Health and Safety Publications Series on Testing and Assessment No. 69. Organisation for Economic Cooperation and Development, Environment Directorate, Paris

8. O'Boyle NM, Banck M, James CA, Morley C, Vandermeersch T, Hutchison GR (2011) Open Babel: An open chemical toolbox. Journal of Cheminformatics 3 (1):33. doi:10.1186/1758-2946-3-33

9. Abraham MH, Chadha HS, Whiting GS, Mitchell RC (1994) Hydrogen bonding. 32. An analysis of water-octanol and water-alkane partitioning and the Δlog p parameter of seiler. J Pharm Sci 83 (8):1085-1100. doi:10.1002/jps.2600830806

10. Brown TN (2022) QSPRs for Predicting Equilibrium Partitioning in Solvent–Air Systems from the Chemical Structures of Solutes and Solvents. J Solution Chem 51 (9):1101-1132. doi:10.1007/s10953-022-01162-2

11. Brown TN (2021) Empirical regressions between system parameters and solute descriptors of polyparameter linear free energy relationships (PPLFERs) for predicting solvent-air partitioning. Fluid Phase Equilib 540:113035. doi:10.1016/j.fluid.2021.113035

12. Admire B, Yalkowsky SH (2013) Predicting the octanol solubility of organic compounds. Journal of pharmaceutical sciences 102 (7):2112-2119. doi:10.1002/jps.23561

13. Brown TN (2022) QSPRs for Predicting Equilibrium Partitioning in Solvent–Air Systems from the Chemical Structures of Solutes and Solvents. Journal of Solution Chemistry. doi:10.1007/s10953-022-01162-2

14. McGowan JC (1984) The estimation of solubility parameters and related properties of liquids. Journal of Chemical Technology and Biotechnology Chemical Technology 34 (1):38-42. doi:<https://doi.org/10.1002/jctb.5040340107>
